# Regulating the Electron Localization of Metallic Bismuth for Boosting CO_2_ Electroreduction

**DOI:** 10.1007/s40820-021-00772-7

**Published:** 2021-12-18

**Authors:** Dan Wu, Renfei Feng, Chenyu Xu, Peng-Fei Sui, Jiujun Zhang, Xian-Zhu Fu, Jing-Li Luo

**Affiliations:** 1 Shenzhen Key Laboratory of Polymer Science and Technology, Guangdong Research Center for Interfacial Engineering of Functional Materials, College of Materials Science and Engineering, Shenzhen University, Shenzhen, 518060 People’s Republic of China; 2 Canadian Light Source Inc., Saskatoon, Saskatchewan, S7N 0X4 Canada; 3 Department of Chemical and Materials Engineering, University of Alberta, Edmonton, AB T6G 1H9 Canada; 4 Institute for Sustainable Energy/College of Science, Shanghai University, Shanghai, 200444 People’s Republic of China

**Keywords:** CO_2_ reduction, Bismuth, Proton transport, Electron localization, Boron

## Abstract

Electrochemical reduction of CO_2_ to formate is economically attractive but improving the reaction selectivity and activity remains challenging. Herein, we introduce boron (B) atoms to modify the local electronic structure of bismuth with positive valence sites for boosting conversion of CO_2_ into formate with high activity and selectivity in a wide potential window. By combining experimental and computational investigations, our study indicates that B dopant differentiates the proton participations of rate-determining steps in CO_2_ reduction and in the competing hydrogen evolution. By comparing the experimental observations with the density functional theory, the dominant mechanistic pathway of B promoted formate generation and the B concentration modulated effects on the catalytic property of Bi are unravelled. This comprehensive study offers deep mechanistic insights into the reaction pathway at an atomic and molecular level and provides an effective strategy for the rational design of highly active and selective electrocatalysts for efficient CO_2_ conversion.

**Supplementary Information:**

The online version contains supplementary material available at 10.1007/s40820-021-00772-7.

## Highlights

- B atoms modify the local electronic structure of Bi with positive valence sites. B doped Bi boosts highly efficient electroreduction of CO_2_ to formate.
- B dopant differentiates the proton participations in CO_2_ RR and HER processes.
- The dominant mechanistic pathway of B promoted formate generation is unraveled.

**Supplementary Information:**

The online version contains supplementary material available at 10.1007/s40820-021-00772-7.

## Graphical abstract

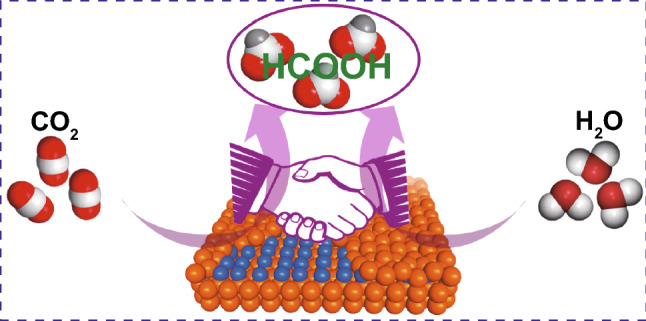

## Introduction

Electrochemical CO_2_ reduction reaction (CO_2_RR) provides a promising approach to the conversion of CO_2_ waste to high-value carbon-based fuels and feedstocks when powered by renewable energy sources, thereby presenting an attractive route to energy and environmental sustainability [1–4]. Among the possible CO_2_RR chemical products, formate (or formic acid) is regarded as an attractive liquid product. It can be used as an important raw material in chemical and pharmaceutical industries, or directly as a hydrogen carrier for fuel cell applications [5–7]. Based on the techno-economic analysis, formate is suggested to be one of the most economically profitable products at industrial scale [8, 9]. Unfortunately, high faradic efficiency can usually be achieved only at the expense of low current density and then would quickly deteriorate with increasing cathodic potentials. As a result, high formate selectivity is limited to a very narrow negative potential window. Therefore, achieving high selectivity combined with high activity in a large potential range is of critical significance to catalyze CO_2_ to formate in the direction of its commercial viability.

Metal surface has been intensively explored in terms of its electroactivity for formate production via CO_2_RR [10–12]. The electrochemical conversion of CO_2_ to formate can be generally considered to start with adsorption of CO_2_ molecules on the metal surface, followed by consecutive proton addition steps to form reaction intermediates and eventually desorbed from the catalyst surface. The key design strategy for a formate-producing metal surface should enable a good control of key intermediates so that the entire reaction pathway has thermodynamically minimum energy barriers. In addition, hydrogen evolution reaction (HER) ineluctably occurs as an additional competing reaction to produce unwanted hydrogen by-products. However, the energetically favorable formate pathway accompanied with hydrogen suppression is difficult to achieve on a clean single metal surface due to the limitation of scaling relationship [13]. Given that the formate generation is governed by the geometric configuration and interfacial chemical bonding of substrate molecules [14–16], controllable modification of the atomic and electronic structure of the metal surface can facilitate CO_2_ adsorption and activation, thereby enhancing electrocatalytic performance. Fortunately, this can potentially be achieved through several strategies including the implantation of alien atoms into metal structures [17–20]. Heteroatom introduction usually redistributes the electron state of metal catalysts [21, 22], thereby regulating the adsorption of reaction intermediates, which intrinsically affect the electrocatalytic performance in CO2RR.

Bismuth (Bi), as a formate-producing electrocatalyst, has drawn insensitive attention due to its earth abundance, low cost and environmental friendliness. Specially, metal Bi has a unique layered structure with an intralayer Bi − Bi bond length of 0.307 nm and an interlayer Bi − Bi bond length of 0.394 nm. This open crystalline structure with large layer distance endows Bi with heteroatom intercalation that decides its unique properties. In this regard, boron (B) appears to be an ideal non-metal candidate to be incorporated into the Bi lattice to induce atomic variation of the neighboring metal sites and uneven charge distribution [18, 23], thus open the possibility of further tuning the electrochemical performances to harvest high selectivity in CO_2_RR process.

In this work, B is intercalated on Bi to realize a 3D porous architecture with abundant active sites, achieving high selectivity and activity for CO_2_ reduction to formate. With the benefits of the unique architecture fabricated, varying the B dopant concentrations can also precisely adjust electronic states of Bi to obtain various oxidative chemical states. Typically, the optimized B modified Bi (denoted as Bi-B2 with B concentration of 2.3%) exhibits > 90% faradic efficiency for formate generation in a large potential window of 494 mV. To understand the underlying reaction mechanisms, density functional theory (DFT) calculations are conducted to elucidate the superior formate generation pathways and the suppression of the competing HER in terms of key intermediates during CO_2_RR on Bi(012) surface with and without B-doping in the subsurface. Besides, the concentration effect of B dopant on the selectivity and activity of Bi is also investigated by experiments and DFT simulations. This work provides a profound perception for tailoring the electronic property of materials, and also lays out an effective strategy to develop highly active and selective CO_2_RR electrocatalysts toward desirable products, especially at more negative potentials.

## Experimental Section

### Preparation of Catalysts

B-doped Bi samples were prepared by a facile one-step process. Since B solubility in Bi is low, Bi(NO_3_)_3_ was added into highly concentrated NaBH_4_ solution instantly in order to alloy the B with Bi at as high loading as possible [24]. First, NaBH_4_ aqueous solution was prepared in an ice bath. Next, 2 mL ethylene glycol (EG) containing Bi(NO_3_)_3_∙5H_2_O was injected rapidly into the NaBH_4_ (5 M, 2 mL) solution until no bubbles formed in the ice bath. The precipitates obtained were repeatedly washed with ethanol to remove the unreacted precursors and other possible byproducts. Finally, the powder was freezing-dried for use. Different amounts of Bi(NO_3_)∙5H_2_O (namely, 600 mg for Bi-B1, 500 mg for Bi-B2, 300 mg for Bi-B3 and 100 mg for Bi-B4) were used. The control sample 500 mg for Bi-NH was synthesized following a similar procedure but using an equal amount of hydrazine hydrate instead of NaBH_4_ as the reducing reagent.

### Characterizations

The morphology of the samples was characterized by field-emission scanning electron microscopy (SEM, SU-70, Hitachi) with accelerating voltages of 5 kV equipped with energy dispersive X-ray spectroscopy (EDS) and high-resolution transmission scanning electron microscopy (HRTEM JEM-F200) with an accelerating voltage of 200 kV equipped with EDS. The phase of catalysts was determined by the SmartLab X-ray diffraction (XRD) with Cu Kα radiation in the range of 2θ from 20° to 80° with step size of 0.01° at a scanning speed of 5° min^−1^. X-ray photoelectron spectroscopy (XPS) was conducted on a Thermo Scientific™ K-Alpha™^+^ spectrometer equipped with a monochromatic Al Kα X-ray source (1486.6 eV) operating at 100 W. All peaks were calibrated with C1s peak binding energy at 284.8 eV. Elemental analysis was carried out by inductively coupled plasma optical emission spectroscopy (ICP-OES, ICAP 7000 SERIES Thermo) with three replicates. The nitrogen adsorption/desorption isotherm and the room-temperature CO_2_ adsorption isotherm were measured by a Micromeritics 3Flex equipment Tristar II gas adsorption analyzer. The Bi L-edge spectra were collected at the 06ID-1 hard X-ray microprobe beamline from Canadian Light Source.

### Preparation of Cathode Electrodes

The catalyst ink was prepared by ultrasonic dispersion of 5 mg of the sample powder with 5 μL Nafion solution (5%) in 950 μL ethanol for 1 h. Next, the as-prepared ink was air-brushed driven by N_2_ gas on the carbon paper with a surface area of 1 × 1 cm^2^. The mass loading was about 0.8 mg cm^−2^.

### Electrochemical Measurements

All CO_2_ reduction experiments were performed in a gas-tight two-compartment H-cell with an ion exchange membrane (Nafion117) as the separator in the middle of the cell. The anode and cathode sides were filled with 40 mL of 0.5 M KHCO_3_, respectively. The Pt foil and Ag/AgCl (saturated KCl) were used as the reference and counter electrodes, respectively. Firstly, the cathode side was electrochemically reduced using the CV method, which ranged from − 0.8 to − 2.0 V (versus Ag/AgCl) at a rate of 0.1 V s^–1^ for 10 cycles to completely reduce the possible oxidized species. The gas products from CO_2_ reduction were analyzed using the gas chromatograph (Fuli 9790Plus) equipped with thermal conductivity and flame ionization detectors on a 3 m-column filled with 5 A molecular sieve column and a packed column (TDX-01, 0.3 m × 4 mm) followed by a methanizer using argon as the carrier gas. The liquid samples were collected and analyzed by ion chromatography (Shenghan, CIC-D120) on an SH-AC-3 anion column (250 × 4.0 mm^2^). The *iR*-corrected potential (versus Ag/AgCl) was converted to RHE using the following equations: *E*_RHE_ = *E*_Ag/AgCl_ + 0.0591 × pH + 0.197.

### Computational Calculations

Density functional theory (DFT) calculations were performed to calculate electronic energies and to optimize geometries using Vienna ab initio Simulation Package (VASP) [25, 26] with the projector augmented wave pseudopotential (PAW) method [27, 28]. The Perdew-Burdew-Ernzerhof (PBE) coupled with van der Waals (vdW) correction [29] was carried to provide an accurate prediction of chemisorption energies. The cutoff energy for the plane wave basis was set to 400 eV. The Bi(012) surface was modeled using the optimized lattice constants of hexagonal Bi unit cell of a = 4.569 Å and c = 11.719 Å and a (3 × 1) periodicity in the x, y directions and 3 atomic layers in the z directions, separated by a vacuum layer in the z direction in the depth of 15 Å. This Bi(012) surface contains 54 Bi atoms. Geometry optimizations were performed until the residual force on each atom became less than 0.02 eV Å^−1^. The electronic energy was considered self-consistent when the energy change was smaller than 10^−5^ eV. The gamma point in the Brillouin zone was used for k-point sampling. Free energies for gaseous molecules were treated using the ideal gas approximation at 298.15 K and 1 bar. Using free energies of reaction intermediates, the free energy change in each proton − electron transfer step was calculated utilizing the computational hydrogen electrode (CHE) method, which estimates a chemical potential of protons and electrons from the chemical potential of gaseous H_2_ and applied potential (μ(H^+^  + e^−^) = 1/2[μ(H_2_)] − eU). In addition, we also applied the subsurface B concentrations of 1/18 monolayer (ML), 9/18 ML, 18/18 ML, respectively, in the Bi(012) slab to examine the various concentrations of boron in the B-doped Bi system.

## Results and Discussion

### Boron Dopant Tuned Morphology and Electronic State of Bi Catalyst

**Fig. 1:**
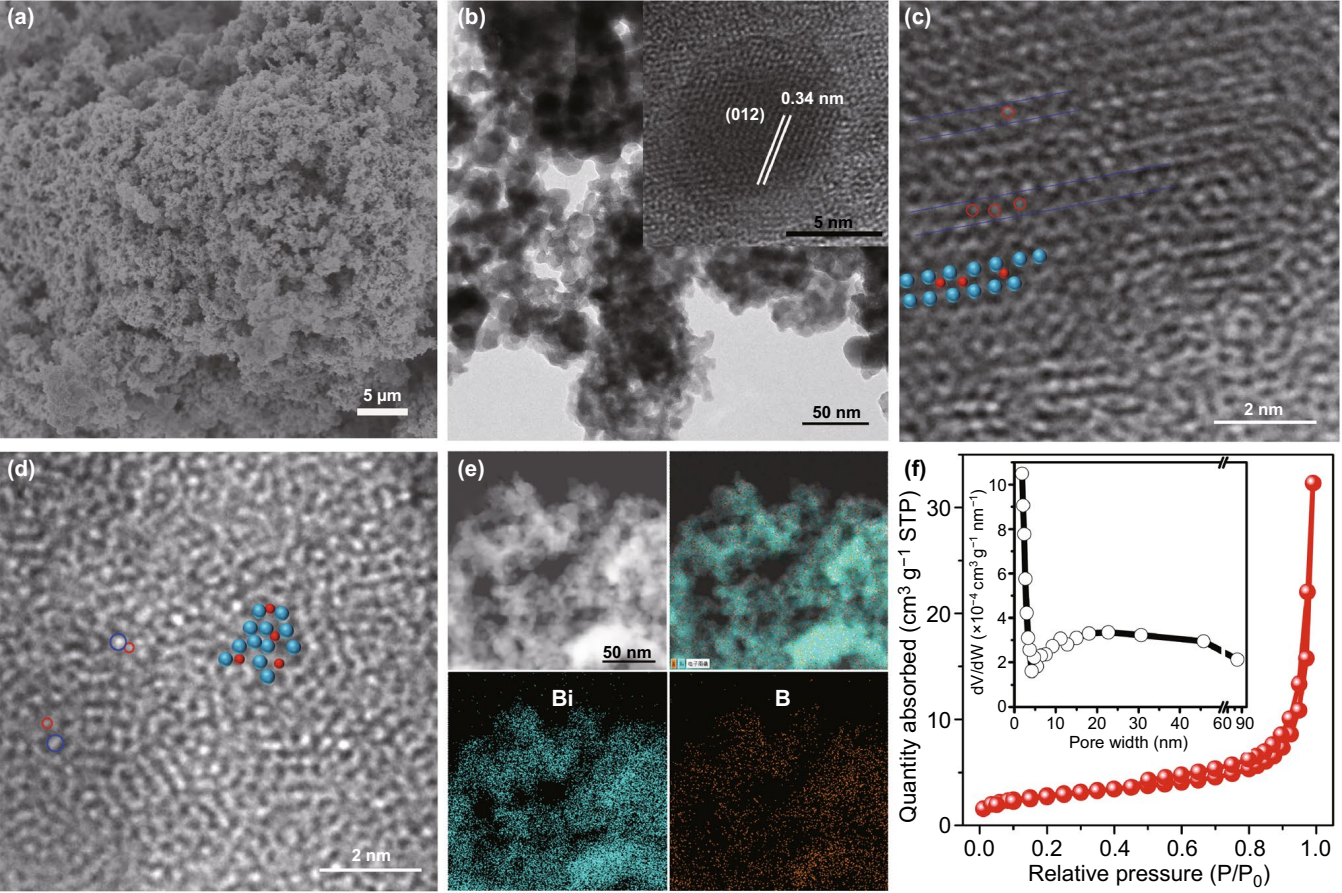
**a** SEM **b** TEM, **c**, **d** HRTEM images (the blue and red balls represent the Bi and B atoms, respectively), **e** HAADF-STEM images and the corresponding STEM-EDS element mappings, **f** N_2_ adsorption–desorption isotherms and the corresponding pore size distribution curve for Bi-B2 catalysts

The rhombohedral Bi has layered structure with the interlayer distance of 0. 394 nm [30] while B has a smaller atomic radius of 0.086 nm, suggesting that the Bi structure can tolerate the incorporation of B atoms. The B-doped Bi was synthesized through a facile wet chemical reduction process using NaBH_4_ and bismuth nitrate as the reducing agent/B source and metal source, respectively. Bi^3+^ salt dissolved in EG was poured into highly concentrated NaBH_4_ solution in order to alloy the B with Bi at as high loading as possible. The BH_4_^−^ ions can reduce the Bi precursor to Bi crystals. Meanwhile, BH_4_^−^ ions may decompose on the surface of Bi nanocrystals to produce B atoms [24]. At this point, B atoms were diffused and successfully inserted into the interstitials of host Bi layered crystal structure [31–33]. For comparison, Bi-H sample was synthesized using hydrazine as the reducing agent to exclude any B doping (Fig. S1). The representative B-doped Bi sample (Bi-B2) is of the rhombohedral crystalline Bi (PDF 01-085-1331) with a dominant (012) diffraction peak (Fig. S2). Compared to Bi-H, the (012) peak of Bi-B2 shifts to low 2θ, corresponding to an enlarged Bi interplanar distance with B incorporation in the interstitials [34, 35]. Due to the large difference of the atomic radius between Bi and B elements, a dramatic variation occurs if B substitutes the Bi metal atoms in lattice position, resulting in a relative unstable structure. Therefore, the B atoms are suggested to thermodynamically occupy the interstitials of Bi metal [36, 37]. Noticeably, the widened diffraction peak for B-B2 suggests that the crystallinity is reduced by B doping [38, 39]. The scanning electron microscopy (SEM) and transmission electron microscopy (TEM) images show that Bi-B2 catalysts have a porous morphology featuring 3D nanostructure on the scale of 5–10 nm (Fig. 1a, b and S3). Some of the Bi nanoparticles demonstrate a clear lattice fringe of 0.34 nm which is assigned to the (012) plane of Bi crystal (insert in Fig. 1b). Besides, B atoms with small atomic diameter are found within the atomic lattice of Bi (Fig. 1c). Notably, the incorporation of Bi also leads to atomic disorder or distortion for a portion of Bi crystals (Figs. 1d and S4). The energy dispersive spectra (EDS) measurement demonstrates that both elemental Bi and B are uniformly distributed both on the nano-scale (Fig. 1e) and on the micrometer scale (Fig. S5). In addition, Bi-B2 also possesses decent porous network, which is comprised of macropores existing in voids between the interconnected fragments as well as mesopores with a broad pore size distribution (Fig. 1f). The SEM images and corresponding EDX mappings of other Bi-B samples are shown in Fig. S6. All the obtained B incorporated Bi samples exhibit a similar 3D porous architecture morphology with pure metallic Bi phase (Fig. S7). The significant figure of merit with porous channels can increase the accessibility of the reactive substrate to the active sites with shortened diffusion distance, which is beneficial for the electrolysis on the catalyst surface [40–42].

**Fig. 2:**
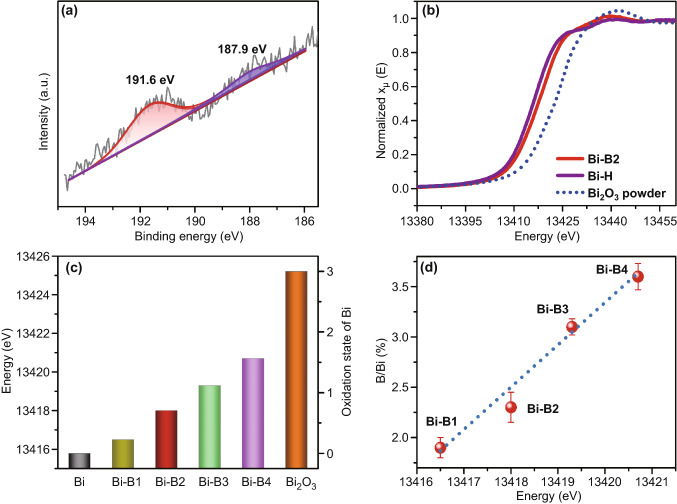
**a** High-resolution XPS B 1s spectra of Bi-B2, **b** Bi L-edge XANES spectra of Bi-B2 and Bi-H samples, **c** oxidation state of Bi and **d** the relationship between the ratio of B/Bi related and the absorption edge position obtained from Bi L-edge XANES in different samples

As shown in Fig. 2a, the X-ray photoelectron spectroscopy (XPS) spectrum of B 1 s located at the binding energy of 191.6 and 187.9 eV can be assigned to the metal-B bond and surface oxidized B, respectively [31, 37, 43]. Moreover, the relative concentration of meal-B in the XPS profiles increases with Ar^+^ etching time (Fig. S8), suggesting that the B is incorporated inside the Bi-B2 architectures instead of only existed on the surface. Noticeably, due to the sponge-like mesoporous structure assembled with small nanosized (5–10 nm) nanoparticles, the vulnerable surface oxidized metallic Bi and B atoms cannot be totally removed on the Ar^+^ etching mode. The X-ray absorption near-edge spectroscopic (XANES) data at the L-edge were recorded to study the impact of boron incorporation on the Bi oxidation state. The absorption edge position of Bi-B2 resides between Bi-H and Bi_2_O_3_ (Fig. 2b), suggesting that B incorporation induces higher oxidation state of Bi than that of Bi-H. This is mainly due to the slightly higher electronegativity of B, allowing electron to easily flow from Bi to adjacent B and leave the surrounding Bi atoms in an electron-deficient state. To visually compare the oxidation state of Bi in Bi-B samples, the Bi oxidation states as a function of L-edge energy shift were acquired (Fig. S9). The energy shifts in Fig. 2c clearly indicate that the average oxidation states of Bi in all the Bi-B architectures vary between 0 and + 3 [44, 45]. The presence of B in the Bi-B samples is also verified by XPS spectra (Fig. S10) and the concentration of B (B/Bi ratio) is quantitatively and qualitatively identified by inductively coupled plasma optical emission spectroscopy (ICP-OES). The atomic ratio of B to Bi in Bi-B samples increases from 1.9% to 3.6% linearly accompanied by more positive shift of the adsorption edge (Fig. 2d), further confirming that B incorporation can tune the oxidation state of Bi in the Bi-B architectures.

### Electrochemical Performances of B Dopant Tuned Bi Catalysts

**Fig. 3:**
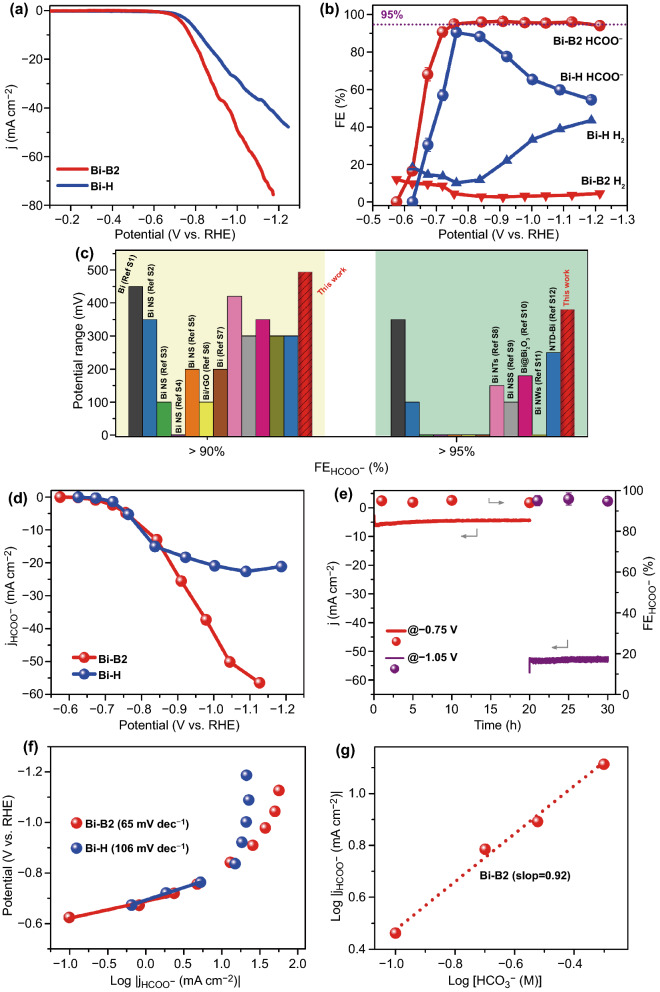
**a** LSV curves in CO_2_ saturated solution and **b** FEs of formate and H_2_ for Bi-B2 and Bi-H, **c** comparison of the-state-of-the-art Bi-based electrocatalysts with high formate FEs, **d** formate partial current density for Bi-B2 and Bi-H, **e** long-term stability test at ‒0.75 V for 20 h and further at ‒1.05 V for 10 h for Bi-B2, **f** Tafel slopes for Bi-B2 and Bi-H and **g** bicarbonate order dependence data at − 0.84 V for Bi-B2

To explore the B incorporation effect on CO_2_RR, the electrochemical CO_2_ reduction measurements for Bi-H and representative Bi-B2 catalysts were conducted in a CO_2_-sarurated 0.5 M KHCO_3_ electrolyte within an H-cell configuration. The electrochemical activity was firstly examined by linear-sweep-voltammetry (LSV) measurements. As shown in Fig. 3a, the LSV curves have the same shape but larger current density below − 0.6 V in CO_2_ atmosphere for Bi-B2, indicating a higher activity of Bi-B2 over Bi-H catalysts. Product analysis demonstrates that H_2_ and negligible CO are the gaseous product and formate is the only liquid product (Figs. 3b and S11). For Bi-B2, the formate is initially detected at as positive as ‒0.57 V, which exhibits smaller overpotential for converting CO_2_ than that of Bi-H with formate initially being measurable at ‒0.67 V. After reaching a maximum value of 90.5% at ‒0.76 V, the formate FE for Bi-H quickly drops to 54.6% at ‒1.18 V with rapidly increased H_2_ selectivity. In contrast, the formate FE for Bi-B2 sharply rises to 90.7% at ‒0.72 V and maintains at > 95.1% in a large potential window of ‒0.75 to ‒1.22 V. A significantly much wider potential window of 0.494 V with formate FE > 90% and 381 mV with formate FE > 95% can be observed from Fig. 3c. To the best of our knowledge, such a wide potential range with high selectivity of formate for Bi-B2 porous architecture outperforms many recently reported state-of-the-art Bi-based electrocatalysts (Table S1). Furthermore, Bi-B2 shows an ultrahigh partial current density of formate up to 56.5 mA cm^−2^ at ‒1.22 V, which is more than twice higher than that of Bi-H (21.1 mA cm^−2^) under the same potential (Fig. 3d). In addition, the double-layer capacitance (C_dl_) value slightly increases from 3.45 mF cm^−2^ of Bi-H to 3.71 mF cm^−2^ of Bi-B2 (Fig. S12). The partial current density of formate was further normalized by C_dl_ value as it is positively correlated to the electrochemical active surface area (ECSA) of the catalysts. The C_dl_-normalized current density indicates that the slightly increased ECSA is not the predominant reason for the enhanced current density after B incorporation. Remarkably, Bi-B2 also exhibits prominently stable electrolysis performance for a long-term test (Fig. 3e). After constantly delivering a current density of about 5 mA cm^−2^ at ‒0.75 V for 20 h, it subsequently keeps a high current density of about 52 mA cm^−2^ at ‒1.05 V for another 10 h. And the corresponding formate FEs stabilize at about 95% with no obvious deterioration. Moreover, the Bi-B2 catalyst also maintains its original morphology after the long-time electrolysis (Fig. S13). Furthermore, the atomic ratio of B/Bi for Bi-B2 is measured as (2.97 ± 0.12)% by ICP-OES analysis, which is comparable to that before long-time electrolysis. The existence of B in the sample is confirmed by the high-resolution B 1 s spectrum with a larger proportion of metal-B bond (Fig. S14a). Owing to the higher electronegativity of B than Bi, Bi would transfer electrons to B in the sample [32, 37]. As a result, more exposed B atoms after electroreduction lead to a positive shift (0.2 eV) of metallic Bi peak (Fig. S14b). Conclusively, the Bi-B2 porous architectures prove to be excellent formate-producing CO_2_RR electrocatalyst with high activity, selectivity, and durability. The reaction kinetics for CO_2_RR is examined by the Tafel plots (Fig. 3f). Bi-B2 demonstrates a smaller Tafel slope of 65 mV dec^−1^ than that of Bi-H (106 mV dec^−1^), evidencing its more favorable CO_2_RR kinetics. As revealed by the Nyquist plots (Fig. S15), the smaller semicircle diameter of Bi-B2 than that of Bi-H implies the much favorable charge transfer kinetics induced by B incorporation [46, 47]. Notably, the Tafel slope close to 118 mV dec^−1^ clarifies that the first electron transfer step is the rate-determining step, whereas the value reaching 59 mV dec^−1^ suggests that the reaction kinetics of CO_2_ reduction is limited by an electron coupling proton (H^+^) transfer step preceded by an initial electron transfer process [48, 49]. The Tafel slopes distinction illustrates that the conversion of CO_2_ to formate is kinetically altered by B incorporation. Given that HCO_3_^‒^ serves as the H^+^ donor in CO_2_ reduction reactions on a thermodynamic basis with the pKa value of HCO_3_^‒^ (pKa = 10.3) being much lower than that of H_2_O (pKa = 14), the hydrogenation effect of HCO_3_^‒^ ranging from 0.1 to 0.5 M is performed [50]. Bi-B2 exhibits a quasi-first-order (0.92) dependence on HCO_3_^‒^ concentration, manifesting that reaction electrokinetic CO_2_ reduction over Bi-B2 is mainly controlled by the H^+^ transfer step. This H^+^ manipulation process can be attributed to the presence of oxidative state of Bi, which is established by the previous observation for the rate-determining route switching [51].

To investigate high-current performance for the practical application of CO_2_RR, we incorporated Bi-B2 catalysts within the flow cell configuration tested in 1 M KOH. As revealed in Fig. S16, the formate selectivity remains above 80% at a wide range and the corresponding partial current density increases from 1.1 to 521.4 mA cm^−2^ as the applied potential changes from ‒0.2 to ‒1.1 V. A large current density at 222 mA cm^−2^ with stable formate selectivity of over 95% is sustained at -0.8 V, indicating an excellent catalytic performance that is suitable for commercial applications.

### Enhanced Reaction Mechanism of B Dopant Tuned Bi Catalysts

To further illustrate the B incorporation enhanced reaction mechanism for formate generation and the origin of high formate selectivity in a wide negative potential range, DFT study was undertaken based on the (012) plane for pristine Bi and B-doped Bi (Bi-B). From the geometrical structure optimization, B with small atomic radius prefers to diffuse into the subsurface of a Bi(012) slab rather than remains on the surface, which is consistent with aforementioned XPS and HRTEM analyses. The formation of HCOOH from CO_2_ involves two proton-coupled electron transfer steps. The CO_2_ absorbed on the catalyst surface (*CO_2_) firstly receives one electron from the electrode and couples one H^+^ from the electrolyte to form formate (*HCOO) or carboxyl (*COOH) intermediate. Then, the intermediates continue to combine an electron and a H^+^ to generate adsorbed HCOOH (*HCOOH) and finally desorb from the catalyst surface to form HCOOH in the electrolyte. As the energy profile in Fig. S17 shown, the lower energy barriers (ΔG) indicate that the energetically favorable reaction pathway from CO_2_ to the formate generation is through the formation of *OCHO intermediate for both pristine Bi and Bi-B. Besides, in situ Raman spectroscopy studies in Fig. S18 clearly evidence the C–O symmetric and asymmetric stretching vibrations of *OCHO (1300 ~ 1600 cm^–1^) and a new band at ∼2900 cm^–1^ as a marker of *HCOO [52–54], further confirming that the conversion of CO_2_ to formate on B doped Bi is through *OCHO intermediates.

**Fig. 4:**
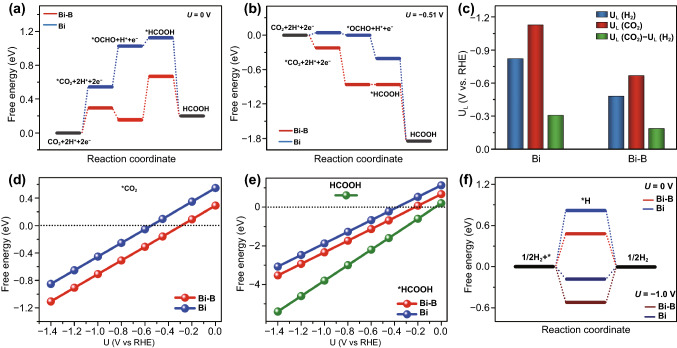
Mechanistic free-energy diagrams at external potential U (versus reversible hydrogen electrode (RHE) at pH 0, 298 K, and 1 atm) of **a** 0 V and **b** -0.51 V, **c** difference in limiting potentials for CO_2_ reduction and H_2_ evolution, free energies of **d** *CO_2_, **e** *HCOOH and **f** *H at different U on pristine Bi (012) and Bi-B (012) surfaces

The free energy diagram of the *OCHO intermediated pathways in terms of multi-step reaction process for the formate generation is plotted in light of the above comparison. At the external potential of 0 V, on the pristine Bi(012) surface, the activation of CO_2_ to form *CO_2_ and the subsequent hydrogenation processes of *CO_2_ to *OCHO and *OCHO to *HCOOH are uphill pathways with large endothermic energy barriers, as shown in Fig. 4a. In the case of Bi-B, the ΔG required to form these key intermediates is much lower compared to those of Bi, indicating a promoted electrocatalytic activity from CO_2_ to formate caused by B incorporation. More specifically, the energy barrier to form *CO_2_ declines markedly from 0.55 eV for Bi to 0.29 eV, implying that CO_2_ molecule is more effectively adsorbed on the Bi-B surface. This agrees well with the fact that the adsorption capacity of Bi-B2 is larger than that of Bi-H from the results of experimental volumetric CO_2_ adsorption isotherms (Fig. S19). Then, the *CO_2_ is exothermically and spontaneously hydrogenated to *OCHO with ΔG = ‒0.13 eV, and *OCHO is further hydrogenated to *HCOOH with an uphill pathway of ΔG = 0.51 eV for Bi-B. In conjunction with the result of kinetic Tafel analysis, the second H^+^-assisted electron transfer process to *HCOOH is considered as the rate-determining step (RDS). Notably, this energy barrier for *OCHO to *HCOOH is diminished when U = ‒0.51 V is applied (Fig. 4b). Even though with the energy barrier close to the thermodynamic minimum, Bi still needs to overcome endothermic free energies with 0.04 eV for *CO_2_ and 0.01 eV for *OCHO to finally obtain HCOOH, demonstrating the difficulty for formate generation at this potential. This is in good agreement with the experimental results that more positive onset potential is needed for Bi-B. Furthermore, the thermodynamic limiting potential difference between CO_2_RR and HER (U_L_(CO_2_)–U_L_(H_2_)) is a descriptor for CO_2_RR selectivity [55, 56]. As shown in Fig. 4c, Bi-B has more positive U_L_(CO_2_)–U_L_(H_2_) value (‒0.19 eV) than pristine Bi (‒0.31 eV), highlighting the advantageous effects of B intercalation on the energetics of formate generation, which is in high consistence with the superior formate selectivity of B-doped Bi, observed from the experimental results.

Given that B doped Bi architectures demonstrate high formate selectivity (FE > 95.1%) in a wide potential range (‒0.75 ~ ‒1.22 V), we sought to elaborate the potential dependent mechanistic insights. As revealed by Fig. S20, the free-energy pathway from CO_2_ to HCOOH becomes a thermodynamical downhill with the potential of more negative than ‒0.6 V applied on Bi and Bi-B surfaces. In majority of the cases, the insufficient supply of active CO_2_ on the catalyst surface accounts for the rapid decrease in selectivity toward target reduction product with increasing cathodic potential [57]. As seen from Fig. 4d, the more negative free energies of *CO_2_ for Bi-B than that for Bi at the same potential suggest that CO_2_ molecules are energetically favorable to bind onto the Bi surface with B incorporation. Furthermore, the smaller energy barrier changes from *HCOOH to HCOOH for Bi-B (Fig. 4e) imply that the *HCOOH can be exothermically easier desorbed from the Bi-B surface to form stable formate liquid product in the electrolyte. Synergically, Bi-B exhibits superior formate selectivity in comparison with Bi at more negative potentials. From the perspective of *OCHO and *HCOOH involvement, it is noteworthy that H^+^ indispensably participates in the hydrogenation process for the conversion of CO_2_ to formate. We have further computationally calculated the energy diagram in terms of the key *H intermediate (Fig. 4f). Interestingly, the free energy of *H is 0.48 eV on Bi-B, significantly lower than on pure Bi (0.82 eV), indicating a higher water dissociation activity on the B modulated surface [58]. At higher cathodic potentials in CO_2_RR, the H^+^ is considered to be supplied by H_2_O dissociation [59–61]. When the binding energy of *H intermediate is too strong, the *H will occupy the active sites on the catalyst surface. However, if the *H is bonded too weakly, it is hard for *H to absorb and to be activated. For instance, with the applied cathodic potential of ‒1.0 V, Bi-B has a larger negative ΔG of ‒0.52 eV than Bi (‒0.18 eV), indicating that it is thermodynamically easier for water dissociation on Bi-B surface to provide H^+^. This sufficient feed of H^+^ ensures the hydrogenation process from *CO_2_ to *HCOOH to proceed in a timely manner on Bi-B surface. Unquestionably, *H intermediates can also form H_2_ from simultaneous HER besides CO_2_RR. However, the free energies of CO_2_ activation to form *CO_2_ and the subsequent two hydrogenation steps are much more negative than that of *H (Fig. S21) in the testing potential range, suggesting that the generation of HCOOH via CO_2_RR is energetically more preferable than via the undesirable HER.

### Effects of B Dopant Concentration on the Electrochemical Performance of Bi Catalysts

**Fig. 5:**
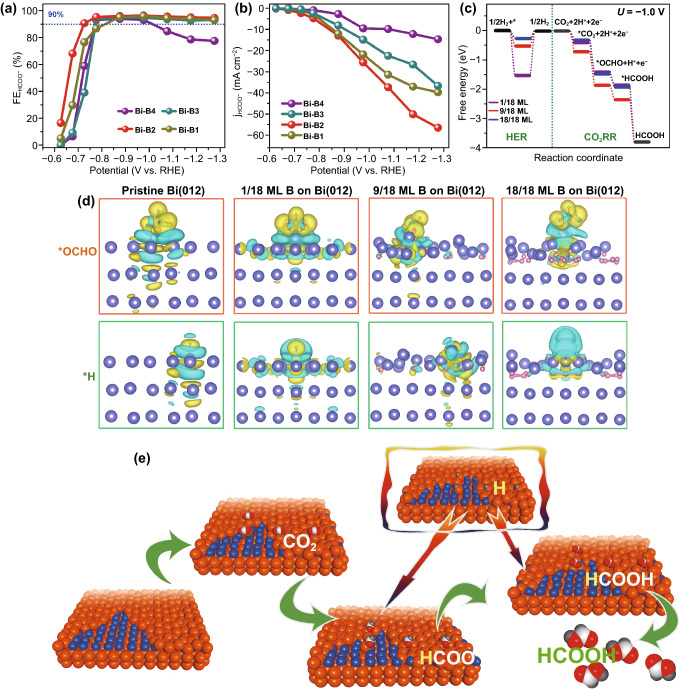
**a** FE and **b** partial current density for formate generation for Bi-B samples, **c** free energy diagrams of Bi with different B concentrations at U = –1.0 V and **d** side view of differential charge densities of *OCHO and *H adsorbed on different surfaces, regions of yellow and cyan denote electron accumulation and depletion, respectively. Blue, pink, brown, red, and pale balls represent Bi, B, C, O, and H atoms, respectively (The value of isosurface is 0.0005 e Å^−3^). **e** Schematic illustration for the H^+^ promoted hydronation process of CO_2_ to formate on Bi surface with B dopant

We also sought to verify whether the B dopant concentration is correlated to the formate selectivity and activity of Bi architecture. As depicted in Fig. 5a, all the B modulated Bi porous architectures show the high formate selectivity. Nevertheless, the FE of formate Bi-B4 with high B concentration (3.6%) gradually decreases when the potential varies from ‒0.7 to ‒1.2 V. Besides, the partial current densities for formate generation on Bi-B1 (B 1.9%) and Bi-B4 are also lower compared to the representative Bi-B2 with B of 2.3% (Fig. 5b). Apparently, Bi porous architectures with B dopant contents of either too low or too high both fall short in achieving high selectivity and activity for formate generation. We initially investigate B substitution at the Bi site or occupancy at an interstitial site in layered Bi lattices with 1/18 monolayer (ML) configuration. On the basis of Fig. S22, the free energy of CO_2_ adsorption, first and second hydrogenation, and *HCOOH desorption for interstitial B doped Bi is lower than that for substitutional B doped Bi, respectively. This result indicates that the interstitial B doping is favored over substitutional B doping in Bi crystals for CO_2_-to-HCOOH conversion. In this study, we have examined the effect of B concentration on the B-doped Bi system with subsurface B concentrations of 1/18 monolayer (ML), 9/18 ML and 18/18 ML to simulate the situations of low, suitable, and high concentrations of B dopant, respectively. As shown in Fig. 5c, the binding energies of the key *CO_2_, *OCHO and *HCOOH intermediates are more positive in the case of 1/18 ML and 18/18 ML configurations than those in the 9/18 ML configuration at U = –1.0 V, inferring a comparably sluggish process to catalyze CO_2_ to formate on Bi with either too low or too many B incorporation. Similar free energy profiles are also observed without external potential applied (Fig. S23). Simultaneously, the exothermal free energies of *H in the 1/18 ML and 9/18 ML configurations are much smaller than those of C-containing intermediates, which guarantees that the formate generation via CO_2_RR is a more favorably achievable process than H_2_ production via competing HER. However, the more positive *H free energy in the 1/18 ML configuration demonstrates that Bi with low concentration of B suffers from a difficult supply of H^+^, thereby affecting the two hydrogenation steps of *CO_2_. Although the free energy of *H in the 18/18 ML configuration is more negative to provide sufficient H^+^ in *CO_2_ hydrogenations, it also favors for the competing H_2_ generation. Moreover, through geometrical optimization, it is found that B-doped Bi surface with the 18/18 ML concentration of B brings about the disordered Bi atomic structure and aggregation of B atoms (Fig. S24). In addition, Fig. S25 exhibits that the charge transfer ability is weaken when increasing B to too high concentration within Bi, which is probably attributed to the lower conductivity induced by the presence of too many B dopants. Consequently, the theoretical results provide solid evidence that Bi with optimized B concentration can achieve the best activity and selectivity for formate formation in CO_2_RR process.

The differential charge densities of *OCHO and *H for subsurface Bi sites without B and with different contents of B were also calculated and shown in Figs. 5d and S26. Compared to the pristine surface, there is evident redistribution of charge density on the B doped Bi surface. The electron depletion is observed on the Bi sites while the neighboring B atoms are surrounded by electron accumulation region, indicating a more positive charged Bi in Bi-B cases. These positively charged Bi sites could serve as an anchor for CO_2_ activation and stabilization. Noticeably, more surface Bi sites are affected and positively charged with increasing B dopants, signifying that more reactive sites participate in the reduction of CO_2_ to formate. With respect to the Bi sites, the strong charge interaction between OCHO and H atom was also partitioned by electron accumulation and depletion region. In the 18/18 ML configuration, obvious charge redistribution is observed among B agglomerations, which severely hinders the electron mobility in the catalyst and reduce the charge transfer rate at the interface [62]. All the above analyses provide solid evidences to elaborate the outstanding electrocatalytic performance of Bi modulated by B surface engineering with optimized B content for value-added formate generation.

Based on the above theoretical calculation and experiment results, the mechanism of CO_2_RR on Bi-B is proposed (Fig. 5e): the subsurface B dopant with optimized concentration on Bi surface is proposed to serve as the promoter in CO_2_ activation and the subsequent protonation of carbon-containing species to transfer CO_2_ to formate. The B intercalation induced positively charged Bi sites favor for the Lewis acid CO_2_ anchoring. With the advantage of porous structure, the CO_2_ molecules are preferably absorbed on the B modified Bi surface. The B introduction also reduces the thermodynamical energy barriers of C-containing intermediates from CO_2_ to formate. At the same time, the surface-adsorbed H* can be easily derived from the near-surface HCO_3_^−^ anions attracted by the positive Bi sites at kinetically controlled low negative potentials, and also from H_2_O molecules with low energy barriers at high negative potentials. The readily accessible supply of *H accelerates the protonation of *CO_2_ to form *OCHO and *HCOO. As a result, the optimized content of B boosts electrochemical CO_2_ reduction to formate on Bi sites with high selectivity and activity over wide potential range.

## Conclusions

In summary, a facile method is developed to prepare B modulated Bi architecture with 3D porous feature. The electroreduction of CO_2_ to formate and its link to Bi catalysts with different B concentrations are theoretically and experimentally studied. The finite elemental analyses including XPS and XANES demonstrate that B dopant leads to positively charged Bi sites. The optimized Bi-B2 sample exhibits a significantly wide potential window of 494 mV with formate FE > 90%. Besides, Bi-B2 shows an ultrahigh partial current density of 56.5 mA cm^−2^ with FE of 95.1% at ‒1.22 V for formate generation. DFT calculation reveals that the subsurface intercalated B reduces the energy barriers of CO_2_ adsorption to form *CO_2_, hydrogenation to form *OCHO and *HCOOH, and desorption of HCOOH from the Bi surface. Furthermore, the absorbed *H species are confirmed to be responsible for the favorable protonation of *CO_2_ to *OCHO and subsequent *HCOOH. With kinetic and thermodynamic preference for CO_2_ transformation and HER suppression, formate is generated with high selectivity in a wide potential range on B intercalated Bi porous architecture with optimized concentration. In addition, a comprehensive mechanistic study also verifies the relationships between the concentration of B dopant and CO_2_RR performance of Bi catalysts. This work opens up more possibilities for rational design of highly efficient CO_2_RR electrocatalysts using earth abundant, cost effective and environmentally friendly materials.

## Supplementary Information

Below is the link to the electronic supplementary material.

Supplementary file1 (PDF 2964 kb)

## References

[1] Ren WH, Zhao C (2020). Paths towards enhanced electrochemical CO_2_ reduction. Natl. Sci. Rev..

[2] Liu HL, Zhu YT, Ma JM, Zhang ZC, Hu WP (2020). Recent advances in atomic-level engineering of nanostructured catalysts for electrochemical CO_2_ reduction. Adv. Funct. Mater..

[3] F. Franco, C. Rettenmaier, H.S. Jeon, B. Roldan Cuenya, Transition metal-based catalysts for the electrochemical CO_2_ reduction: From atoms and molecules to nanostructured materials. Chem. Soc. Rev. **49**(19), 6884–6946 (2020). Doi: 10.1039/d0cs00835d 10.1039/d0cs00835d 32840269

[4] Lu S, Shi Y, Meng N, Lu S, Yu Y (2020). Electrosynthesis of syngas via the co-reduction of CO_2_ and H_2_O. Cell Rep. Phys. Sci..

[5] Tao ZX, Wu ZS, Wu YS, Wang HL (2020). Activating copper for electrocatalytic CO_2_ reduction to formate via molecular interactions. ACS Catal..

[6] Xiong Y, Dong J, Huang ZQ, Xin P, Chen W (2020). Single-atom Rh/N-doped carbon electrocatalyst for formic acid oxidation. Nat. Nanotechnol..

[7] Zhao S, Li S, Guo T, Zhang S, Wang J (2019). Advances in Sn-based catalysts for electrochemical CO_2_ reduction. Nano Micro Lett..

[8] Bushuyev OS, De Luna P, Dinh CT, Tao L, Saur G (2018). What should we make with CO_2_ and how can we make it?. Joule.

[9] Spurgeon JM, Kumar B (2018). A comparative technoeconomic analysis of pathways for commercial electrochemical CO_2_ reduction to liquid products. Energy Environ. Sci..

[10] N. Han, P. Ding, L. He, Y.Y. Li, Y.G. Li, Promises of main group metal-based nanostructured materials for electrochemical CO_2_ reduction to formate. Adv. Energy Mater. **10**(11), (2020). Doi: 10.1002/aenm.201902338

[11] Ding P, Zhao HT, Li TS, Luo YS, Fan GY (2020). Metal-based electrocatalytic conversion of CO_2_ to formic acid/formate. J. Mater. Chem. A.

[12] Wu D, Wang XW, Fu XZ, Luo JL (2021). Ultrasmall bi nanoparticles confined in carbon nanosheets as highly active and durable catalysts for CO_2_ electroreduction. Appl. Catal. B Environ..

[13] Z.W. Seh, J. Kibsgaard, C.F. Dickens, I. Chorkendorff, J.K. Norskov et al., Combining theory and experiment in electrocatalysis: Insights into materials design. Science **355**(6321), eaad4998 (2017). Doi: 10.1126/science.aad4998 10.1126/science.aad4998 28082532

[14] Xie CL, Niu ZQ, Kim D, Li MF, Yang PD (2020). Surface and interface control in nanoparticle catalysis. Chem. Rev..

[15] Chen K, Qi K, Zhou T, Yang T, Zhang Y (2021). Water-dispersible CsPbBr_3_ perovskite nanocrystals with ultra-stability and its application in electrochemical C_O_2 reduction. Nano Micro Lett..

[16] Pan Z, Han E, Zheng J, Lu J, Wang X (2020). Highly efficient photoelectrocatalytic reduction of CO_2_ to methanol by a P-N heterojunction CeO_2_/CuO/Cu catalyst. Nano Micro Lett..

[17] Wu Y, Zhai P, Cao S, Li Z, Zhang B (2020). Beyond d orbits: Steering the selectivity of electrochemical CO_2_ reduction via hybridized sp band of sulfur-incorporated porous cd architectures with dual collaborative sites. Adv. Energy Mater..

[18] Jiang B, Zhang XG, Jiang K, Wu DY, Cai WB (2018). Boosting formate production in electrocatalytic CO_2_ reduction over wide potential window on Pd surfaces. J. Am. Chem. Soc..

[19] Cheng H, Liu S, Zhang J, Zhou T, Zhang N (2020). Surface nitrogen-injection engineering for high formation rate of CO_2_ reduction to formate. Nano Lett..

[20] Zheng XL, De Luna P, de Arquer FPG, Zhang B, Becknell N (2017). Sulfur-modulated tin sites enable highly selective electrochemical reduction of CO_2_ to formate. Joule.

[21] Wu ZZ, Gao FY, Gao MR (2021). Regulating the oxidation state of nanomaterials for electrocatalytic CO_2_ reduction. Energy Environ. Sci..

[22] Xue D, Xia H, Yan W, Zhang J, Mu S (2020). Defect engineering on carbon-based catalysts for electrocatalytic CO_2_ reduction. Nano Micro Lett.

[23] Zhou Y, Che F, Liu M, Zou C, Liang Z (2018). Dopant-induced electron localization drives CO_2_ reduction to C_2_ hydrocarbons. Nat. Chem..

[24] Carenco S, Portehault D, Boissiere C, Mezailles N, Sanchez C (2013). Nanoscaled metal borides and phosphides: recent developments and perspectives. Chem. Rev..

[25] Kresse G, Furthmuller J (1996). Efficiency of ab-initio total energy calculations for metals and semiconductors using a plane-wave basis set. Comput. Mater. Sci..

[26] Kresse G, Furthmuller J (1996). Efficient iterative schemes for ab initio total-energy calculations using a plane-wave basis set. Phys. Rev. B.

[27] Blochl PE (1994). Projector augmented-wave method. Phys. Rev. B.

[28] Kresse G, Joubert D (1999). From ultrasoft pseudopotentials to the projector augmented-wave method. Phys. Rev. B.

[29] Grimme S, Antony J, Ehrlich S, Krieg H (2010). A consistent and accurate ab initio parametrization of density functional dispersion correction (DFT-D) for the 94 elements H-Pu. J. Chem. Phys..

[30] Lei KX, Wang CC, Liu LJ, Luo YW, Mu CN (2018). A porous network of bismuth used as the anode material for high-energy-density potassium-ion batteries. Angew. Chem. Int. Ed..

[31] Hao W, Yao D, Xu Q, Wang R, Zhang C (2021). Highly efficient overall-water splitting enabled via grafting boron-inserted Fe-Ni solid solution nanosheets onto unconventional skeleton. Appl. Catal. B Environ..

[32] Li Y, Yu H, Wang Z, Liu S, Xu Y (2019). Boron-doped silver nanosponges with enhanced performance towards electrocatalytic nitrogen reduction to ammonia. Chem. Commun..

[33] Khan K, Tareen AK, Aslam M, Sagar RUR, Zhang B (2020). Recent progress, challenges, and prospects in two-dimensional photo-catalyst materials and environmental remediation. Nano Micro Lett..

[34] Chen GR, An J, Meng YM, Yuan CZ, Matthews B (2019). Cation and anion co-doping synergy to improve structural stability of Li- and Mn-rich layered cathode materials for lithium-ion batteries. Nano Energy.

[35] Jiang K, Chang J, Wang H, Brimaud S, Xing W (2016). Small addition of boron in palladium catalyst, big improvement in fuel cell's performance: What may interfacial spectroelectrochemistry tell?. ACS Appl. Mater. Interfaces.

[36] Zhang L, Lu J, Yin S, Luo L, Jing S (2018). One-pot synthesized boron-doped RhFe alloy with enhanced catalytic performance for hydrogen evolution reaction. Appl. Catal. B: Environ..

[37] Lv H, Xu D, Sun L, Henzie J, Suib SL (2019). Ternary palladium-boron-phosphorus alloy mesoporous nanospheres for highly efficient electrocatalysis. ACS Nano.

[38] Zhang G, Xu Y, He C, Zhang P, Mi H (2021). Oxygen-doped crystalline carbon nitride with greatly extended visible-light-responsive range for photocatalytic H_2_ generation. Appl. Catal. B: Environ..

[39] Y. Yang, L. Zhuang, R. Lin, M. Li, X. Xu et al., A facile method to synthesize boron-doped Ni/Fe alloy nano-chains as electrocatalyst for water oxidation. J. Power Sources **349, **68–74 (2017). Doi: 10.1016/j.jpowsour.2017.03.028

[40] Wei F, Wang T, Jiang X, Ai Y, Cui J (2020). Controllably engineering mesoporous surface and dimensionality of SnO_2_ toward high-performance CO_2_ electroreduction. Adv. Funct. Mater..

[41] Wu D, Liu JW, Liang Y, Xiang K, Fu XZ (2019). Electrochemical transformation of facet-controlled bioi into mesoporous bismuth nanosheets for selective electrocatalytic reduction of CO_2_ to formic acid. Chemsuschem.

[42] Zhou M, Lin Y, Xia H, Wei X, Yao Y (2020). A molecular foaming and activation strategy to porous N-doped carbon foams for supercapacitors and CO_2_ capture. Nano Micro Lett..

[43] Zhang X, Kim D, Guo X, Zhu Y, Lee LYS (2021). Impacts of boron doping on the atomic structure, stability, and photocatalytic activity of Cu_3_P nanocrystals. Appl. Catal. B Environ..

[44] Shi Y, Ji Y, Long J, Liang Y, Liu Y (2020). Unveiling hydrocerussite as an electrochemically stable active phase for efficient carbon dioxide electroreduction to formate. Nat. Commun..

[45] Zheng W, Chen F, Zeng Q, Li Z, Yang B (2020). A universal principle to accurately synthesize atomically dispersed metal-N_4_ sites for CO_2_ electroreduction. Nano Micro Lett..

[46] Liang Y, Zhou W, Shi Y, Liu C, Zhang B (2020). Unveiling in situ evolved In/In_2_O_3_ heterostructure as the active phase of In_2_O_3_ toward efficient electroreduction of CO_2_ to formate. Sci. Bullet..

[47] Meng N, Zhou W, Yu Y, Liu Y, Zhang B (2019). Superficial hydroxyl and amino groups synergistically active polymeric carbon nitride for CO_2_ electroreduction. ACS Catal..

[48] Dunwell M, Luc W, Yan YS, Jiao F, Xu BJ (2018). Understanding surface-mediated electrochemical reactions: CO_2_ reduction and beyond. ACS Catal..

[49] Zhang XL, Sun XH, Guo SX, Bond AM, Zhang J (2019). Formation of lattice-dislocated bismuth nanowires on copper foam for enhanced electrocatalytic CO_2_ reduction at low overpotential. Energy Environ. Sci..

[50] Wu D, Huo G, Chen WY, Fu XZ, Luo JL (2020). Boosting formate production at high current density from CO_2_ electroreduction on defect-rich hierarchical mesoporous Bi/Bi_2_O_3_ junction nanosheets. Appl. Catal. B: Environ..

[51] Deng P, Wang H, Qi R, Zhu J, Chen S (2020). Bismuth oxides with enhanced bismuth–oxygen structure for efficient electrochemical reduction of carbon dioxide to formate. ACS Catal..

[52] Ismail AM, Samu GF, Balog A, Csapo E, Janaky C (2019). Composition-dependent electrocatalytic behavior of Au-Sn bimetallic nanoparticles in carbon dioxide reduction. ACS Energy Lett..

[53] Vasileff A, Zhi X, Xu CC, Ge L, Jiao Y (2019). Selectivity control for electrochemical CO_2_ reduction by charge redistribution on the surface of copper alloys. ACS Catal..

[54] W.T. Ichinohe Y, Hatta A, Electrochemical reduction of CO_2_ on silver as probed by surface-enhanced raman scattering. J. Raman Spectrosc. **26**(5), 335–340 (1995). Doi: 10.1002/jrs.1250260503

[55] Jiao L, Yang WJ, Wan G, Zhang R, Zheng XS (2020). Single-atom electrocatalysts from multivariate metal-organic frameworks for highly selective reduction of CO_2_ at low pressures. Angew. Chem. Int. Ed..

[56] Zhang EH, Wang T, Yu K, Liu J, Chen WX (2019). Bismuth single atoms resulting from transformation of metal-organic frameworks and their use as electrocatalysts for CO_2_ reduction. J. Am. Chem. Soc..

[57] Chen ZP, Mou KW, Wang XH, Liu LC (2018). Nitrogen-doped graphene quantum dots enhance the activity of Bi_2_O_3_ nanosheets for electrochemical reduction of CO_2_ in a wide negative potential region. Angew. Chem. Int. Ed..

[58] Ma WC, Xie SJ, Zhang XG, Sun FF, Kang JC (2019). Promoting electrocatalytic CO_2_ reduction to formate via sulfur-boosting water activation on indium surfaces. Nat. Commun..

[59] Nesbitt NT, Burdyny T, Simonson H, Salvatore D, Bohra D (2020). Liquid−solid boundaries dominate activity of CO_2_ reduction on gas-diffusion electrodes. ACS Catal..

[60] Ooka H, Figueiredo MC, Koper MTM (2017). Competition between hydrogen evolution and carbon dioxide reduction on copper electrodes in mildly acidic media. Langmuir.

[61] Liu TY, Diao P, Lin Z, Wang HL (2020). Sulfur and selenium doped nickel chalcogenides as efficient and stable electrocatalysts for hydrogen evolution reaction: The importance of the dopant atoms in and beneath the surface. Nano Energy.

[62] Chen ZP, Zhang XX, Jiao MY, Mou KW, Zhang XP (2020). Engineering electronic structure of stannous sulfide by amino-functionalized carbon: Toward efficient electrocatalytic reduction of CO_2_ to formate. Adv. Energy Mater..

