# Supplementary material for: Regulating the Electron Localization of Metallic Bismuth for Boosting CO2 Electroreduction
Source: Nanomicro Lett. 2021 Dec 18;14:38. doi: 10.1007/s40820-021-00772-7 (PMC8683517; doi:10.1007/s40820-021-00772-7)
Supplement: Supplementary file 1 — Supplementary file1 (PDF 2964 kb) [file 40820_2021_772_MOESM1_ESM.pdf]

Supporting Information for

Regulating the Electron Localization of Metallic Bismuth for Boosting CO_2 Electroreduction

Dan Wu[1], Renfei Feng[2], Chenyu Xu[3], Peng-Fei Sui[3], Jiujun Zhang[4], Xian-Zhu Fu[1,]* and Jing-Li Luo[1,]*

[1]Shenzhen Key Laboratory of Polymer Science and Technology, Guangdong Research Center for Interfacial Engineering of Functional Materials, College of Materials Science and Engineering, Shenzhen University, Shenzhen 518060, P. R. China

[2]Canadian Light Source Inc., Saskatoon, S7N 0X4 Saskatchewan, Canada

[3]Department of Chemical and Materials Engineering, University of Alberta, Edmonton, Alberta T6G 1H9, Canada

[4]Institute for Sustainable Energy/College of Science, Shanghai University, Shanghai 200444, P. R. China

*Corresponding authors. E-mail: xz.fu@szu.edu.cn (Xian-Zhu Fu), jingli.luo@ualberta.ca (Jing-Li Luo)

S1 Experimental Section

S1.1 Flow Cell Measurements

The flow cell measurements were performed within a custom-designed flow cell reactor consisting catalysts loaded gas-diffusion layer (1 cm^2) as the cathode, anion exchange membrane separator and Pt sheet as the anode. These three compositions were positioned and clamped together using polytetrafluoroethylene spacers for electrolyte circulation. Ag/AgCl reference electrode (saturated KCl) was located inside the cathode compartment. During the measurement, the CO_2 gas feed for the reaction was supplied at rate of 20 mL min^{-1} as a continuous stream over the cathodic GDL using a flow controller (D07-19B, Sevenstar). 1M KOH was used as the electrolyte. Both the catholyte and the anolyte streams were circulated through the electrolyte channels using a syringe pump (BT100-2 J, LONGER) at flow rate of 8 mL min^{-1}.

S1.2 Raman Spectroscopy Measurements

The Raman spectra acquisition was carried out using a RENIDHAW invia Raman Microscope and 514 nm excitation laser. A homemade H-type in situ cell was used for Raman signal collection with a piece of round quartz glass on the top of the cell to allow light transmission. A piece of the catalysts supported carbon paper was inserted through the wall of the cell to keep the plane of the working electrode perpendicular to the incident laser. Pt wire was used as a counter electrode and Ag/AgCl (saturated KCl) was used as a reference electrode. A 0.5 M $NaHCO_3$ aqueous solution with CO_2 bubbling continuously was pumped through the cathode compartment. The Raman spectra were then recorded at different potentials that driven by a potential station.

S2 Supplementary Figures and Table

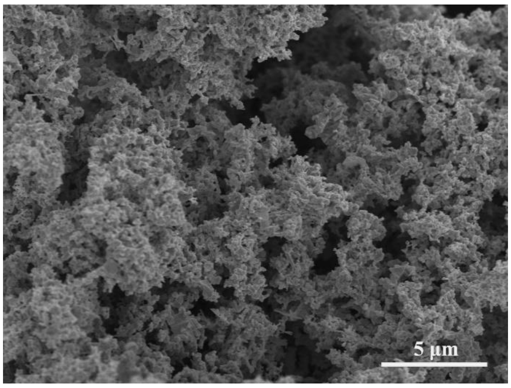

Fig. S1 SEM image of Bi-H sample

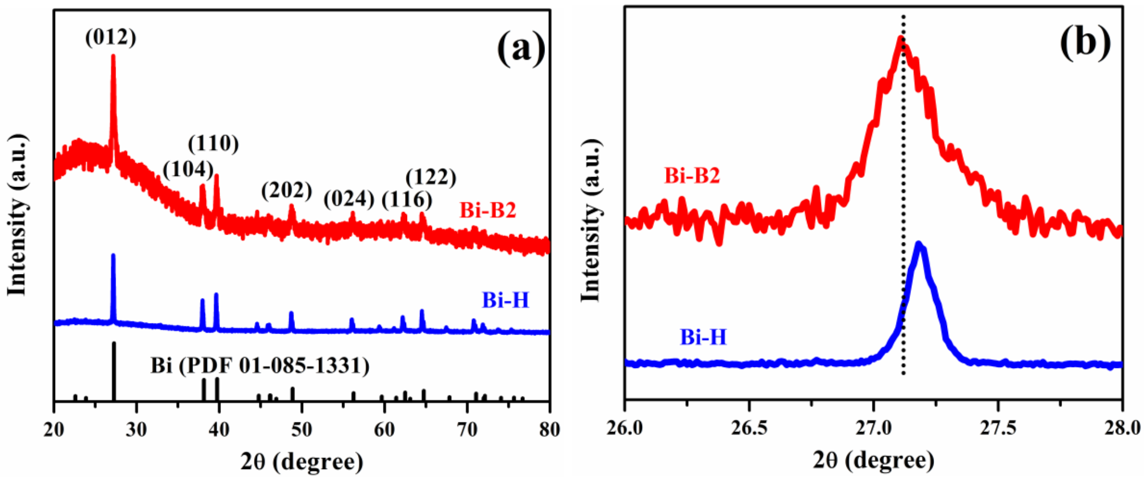

Fig. S2 XRD patterns of (**a**) Bi-H and (**b**) Bi-B2 catalysts

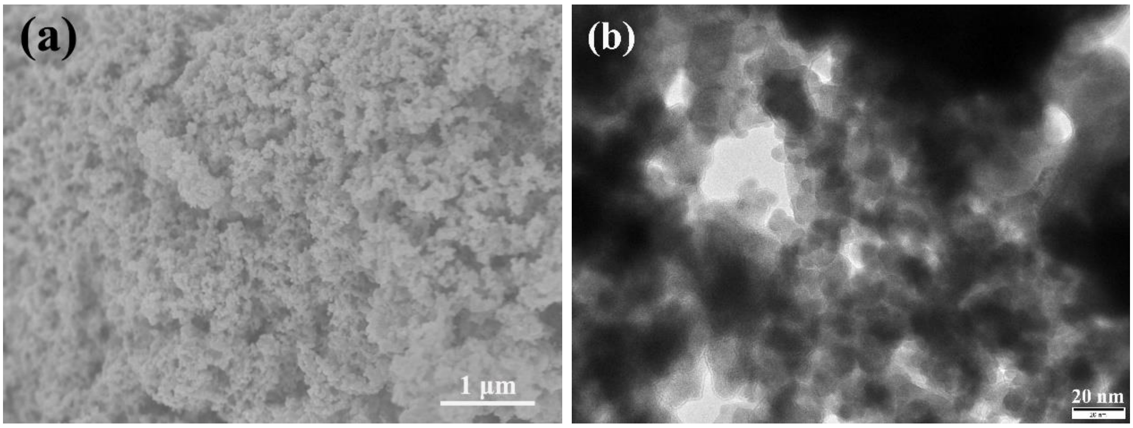

Fig. S3 (**a**) SEM and (**b**) TEM images of Bi-B2 sample

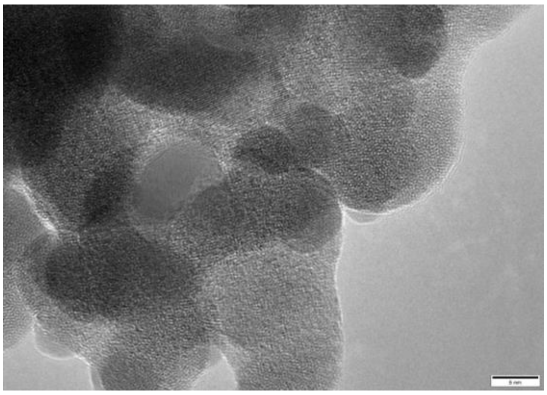

Fig. S4 HRTEM images of Bi-B2 sample

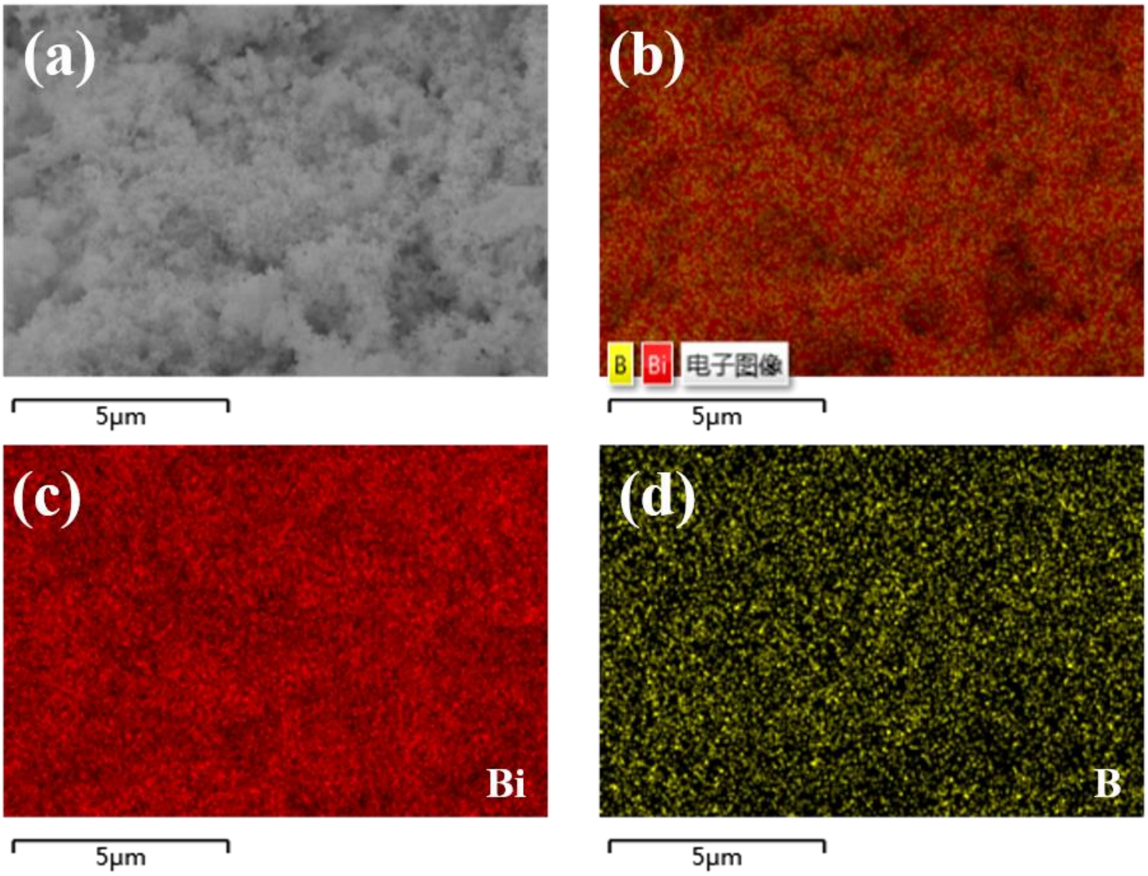

Fig. S5 (**a**) SEM and corresponding elemental mappings of (**b**) overall, (**c**) Bi and (**d**) B for Bi-B2 sample

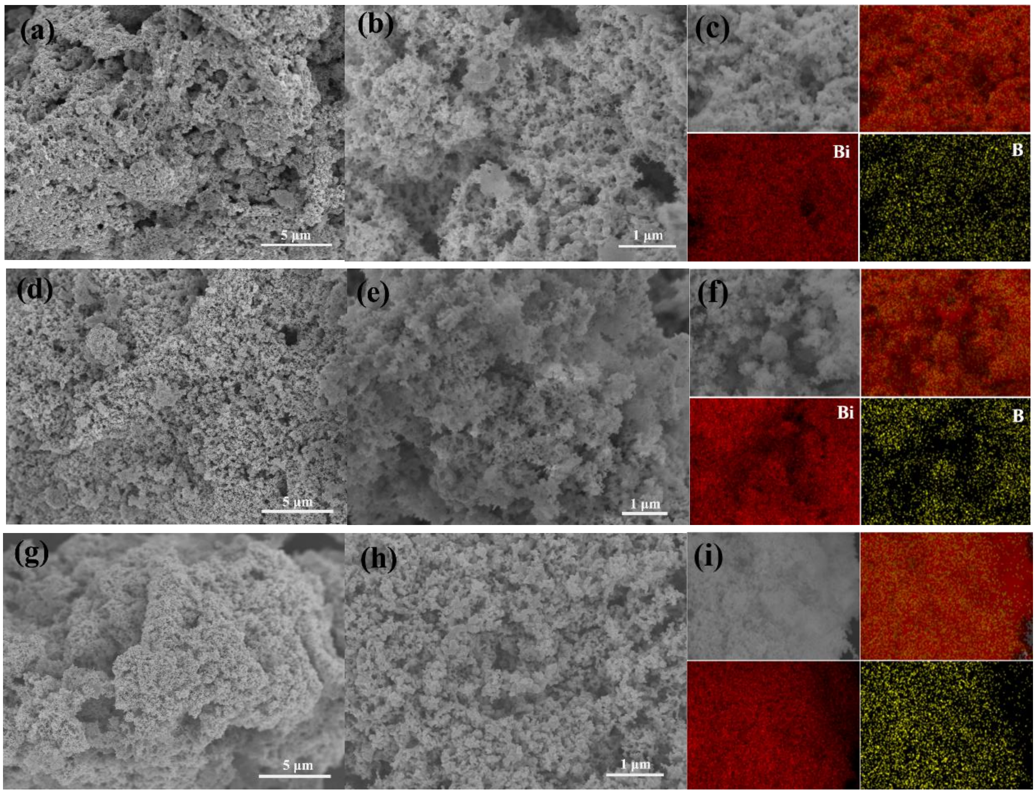

Fig. S6 SEM images of (**a**, **b**) Bi-B4, (**d**, **e**) Bi-B3, (**g**, **h**) Bi-B1 and corresponding EDX mappings of (**c**) Bi-B4, (**f**) Bi-B3 and (**i**) Bi-B1 samples

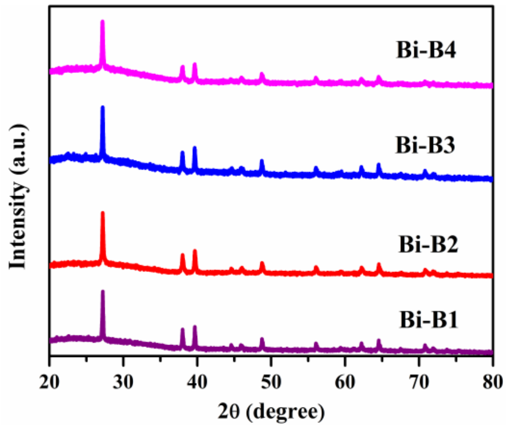

Fig. S7 XRD patterns of Bi-B4, Bi-B3, Bi-B2 and Bi-B1 samples

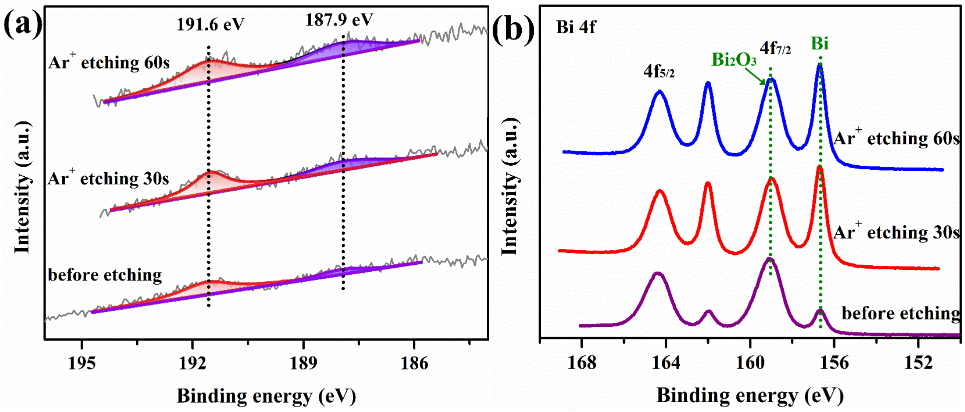

Fig. S8 XPS spectra for (**a**) B 1s and (**b**) Bi 4f of Bi-B2 with Ar^{+} etching

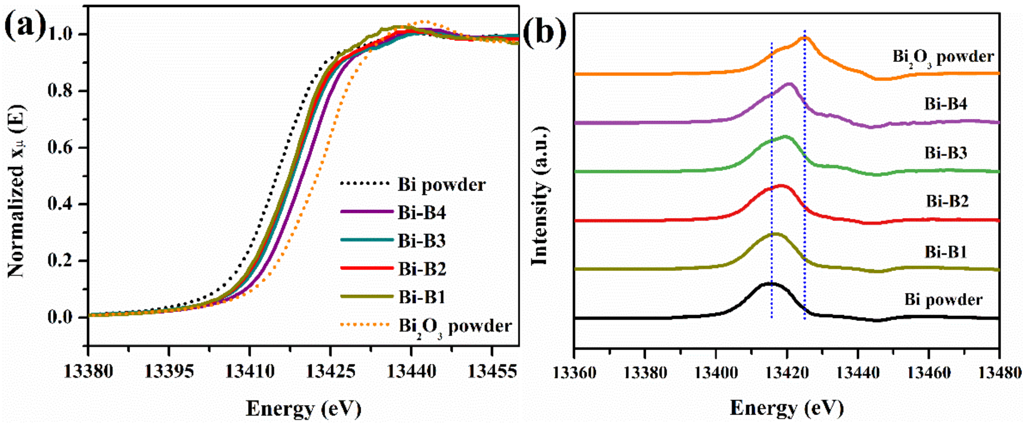

Fig. S9 (**a**) Bi L-edge edge XANES spectra and (**b**) corresponding first derivatives of samples. Commercial Bi and Bi_2O_3 powders are also listed as the references

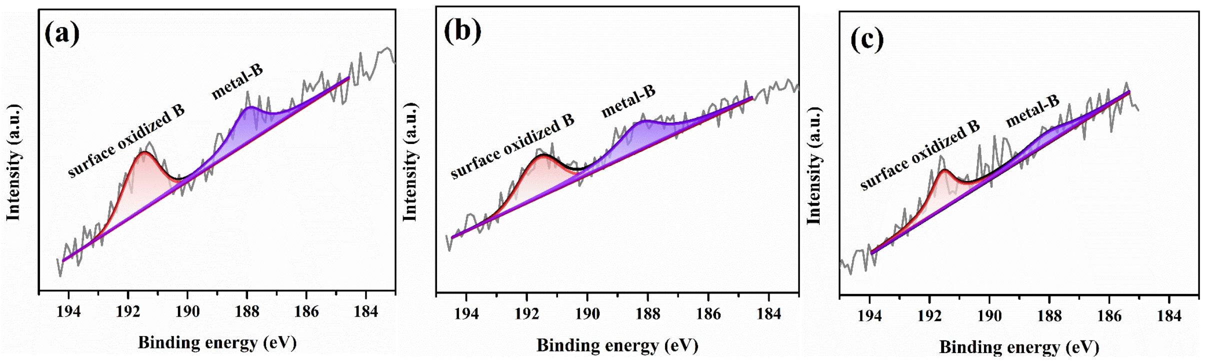

Fig. S10 XPS B 1s spectra of (**a**) Bi-B4, (**b**) Bi-B3 and (**c**) Bi-B1 samples

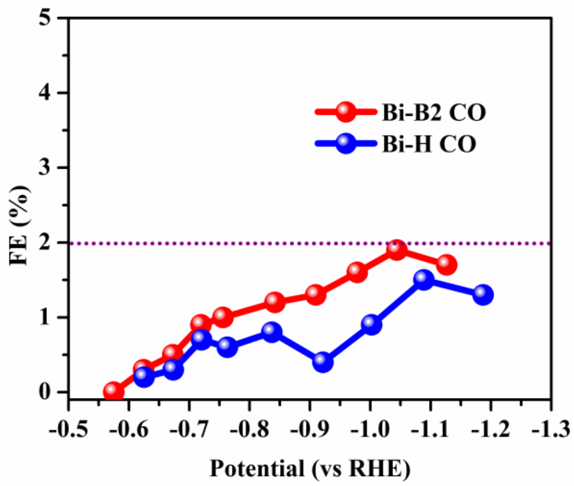

Fig. S11 FEs of CO on Bi-B2 and Bi-H samples

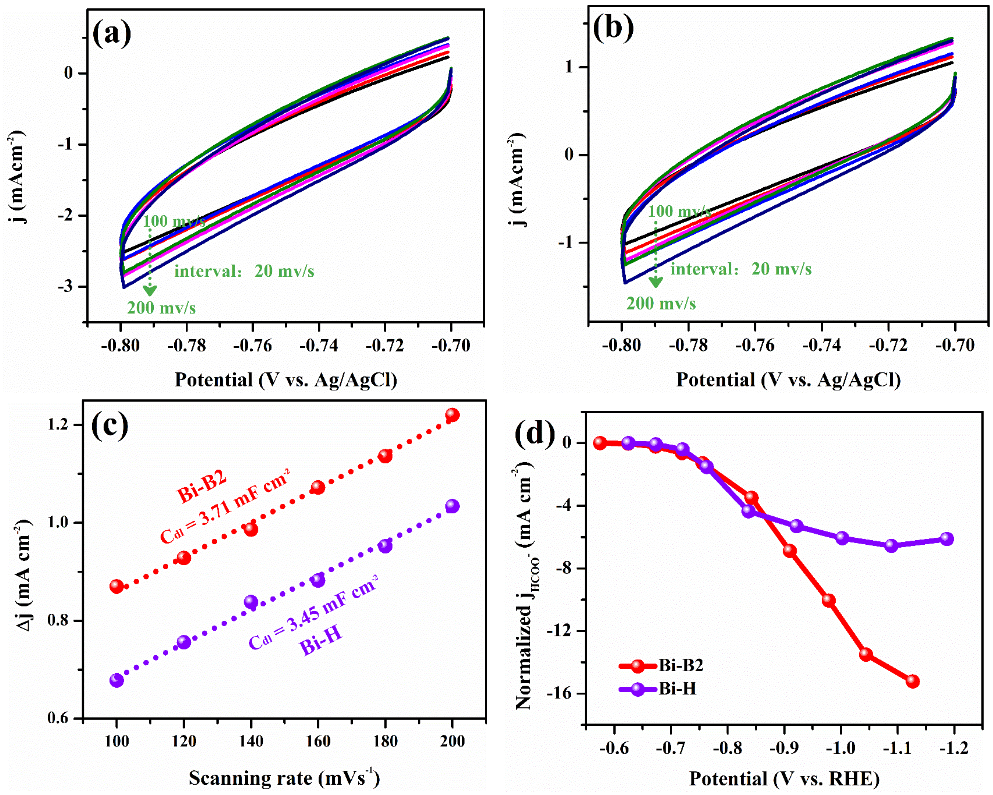

Fig. S12 CV curves for (**a**) Bi-B2 and (**b**) Bi-H, (**c**) corresponding current density differences plotted against scanning rates, and (**d**) normalized formate partial current density

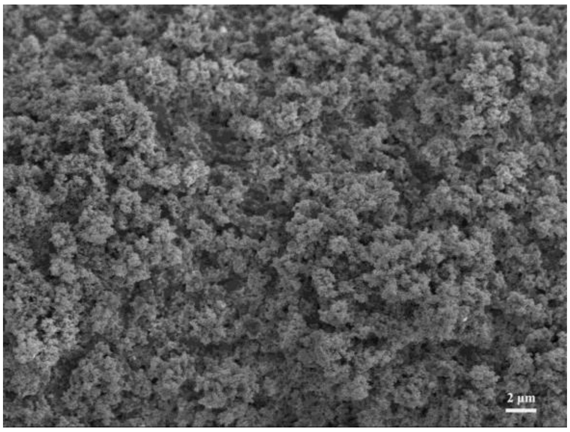

Fig. S13 SEM images of the Bi-B2 catalyst after long-time electrolysis

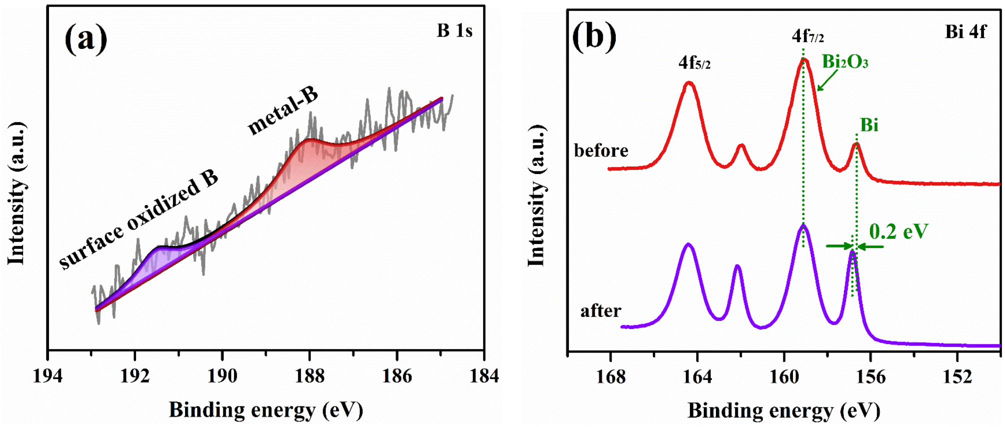

Fig. S14 XPS spectra of (**a**) B 1s and (**b**) Bi 4f of the Bi-B2 catalyst after long-time electrolysis

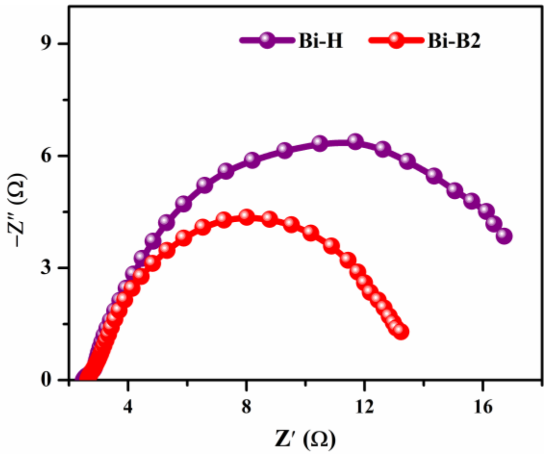

Fig. S15 Nyquist plots of Bi-B2 and Bi-H samples

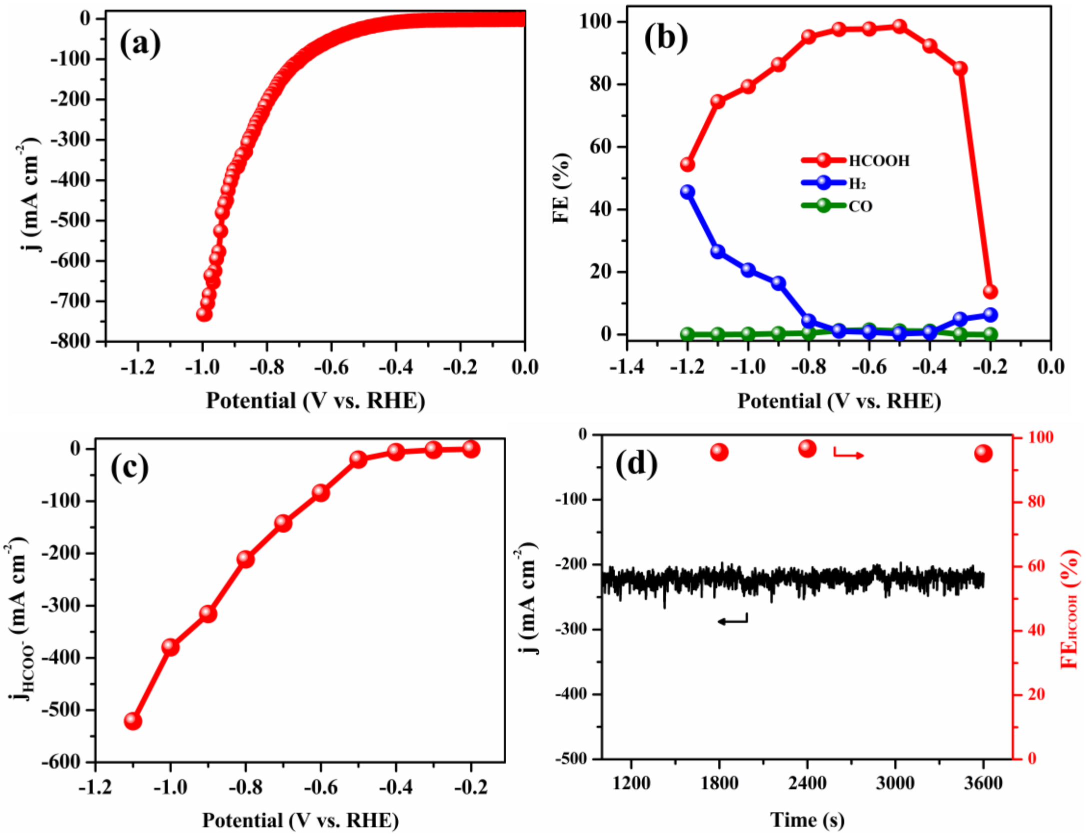

Fig. S16 (**a**) LSV curve, (**b**) FEs of formate, H_2 and CO, (**c**) formate partial current density and (**d**) stability test at -0.8 V (vs RHE) for the Bi-B2 catalysts in the flow cell

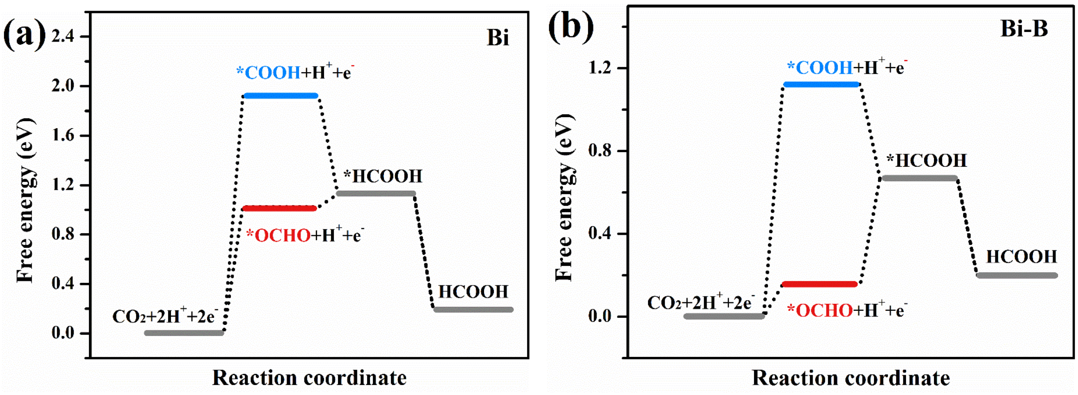

Fig. S17 Free energy diagrams the reaction pathways for the formate generation in terms of two intermediates on the (012) plane for (**a**) Bi and (**b**) Bi-B

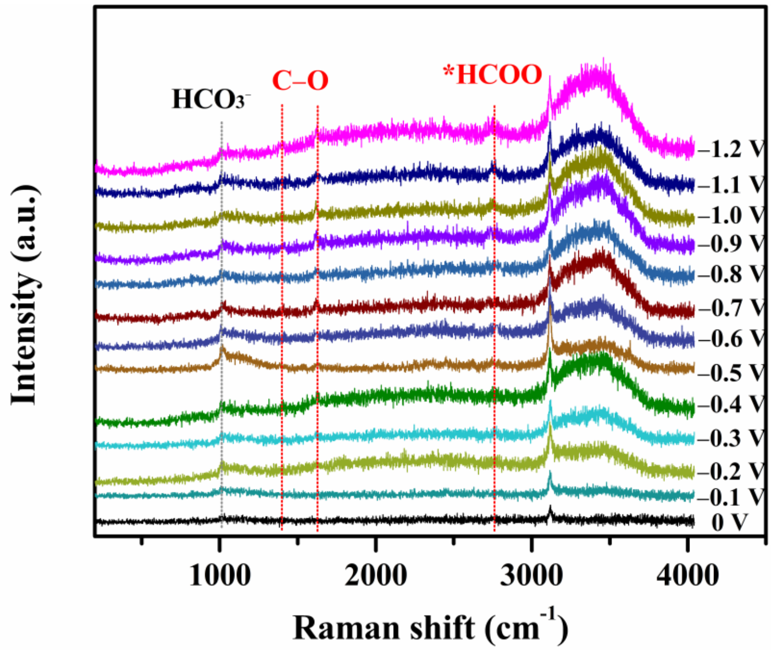

Fig. S18 In situ Raman spectra of Bi-B2 during electrochemical reduction of CO_2 at different potentials (vs RHE) in 0.5 M CO_2-saturated $KHCO_3$

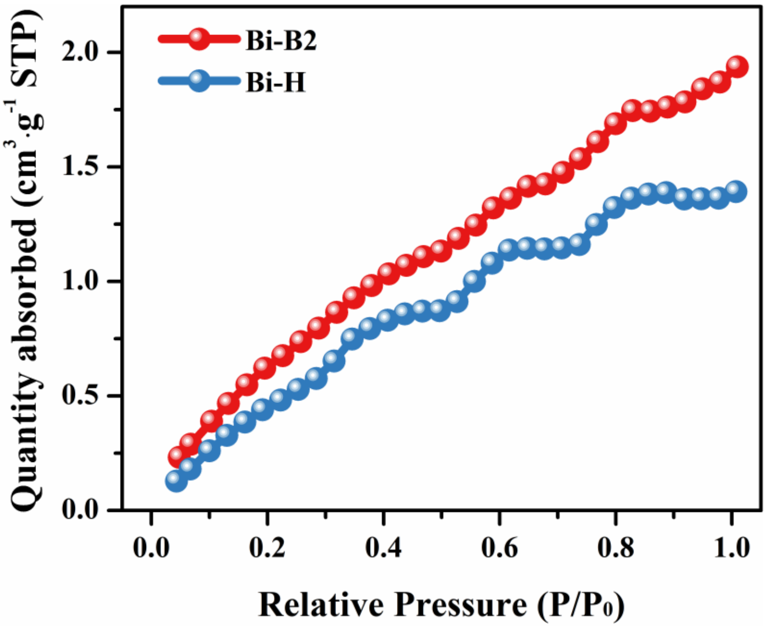

Fig. S19 Room-temperature CO_2 adsorption isotherms for Bi-H and Bi-B2

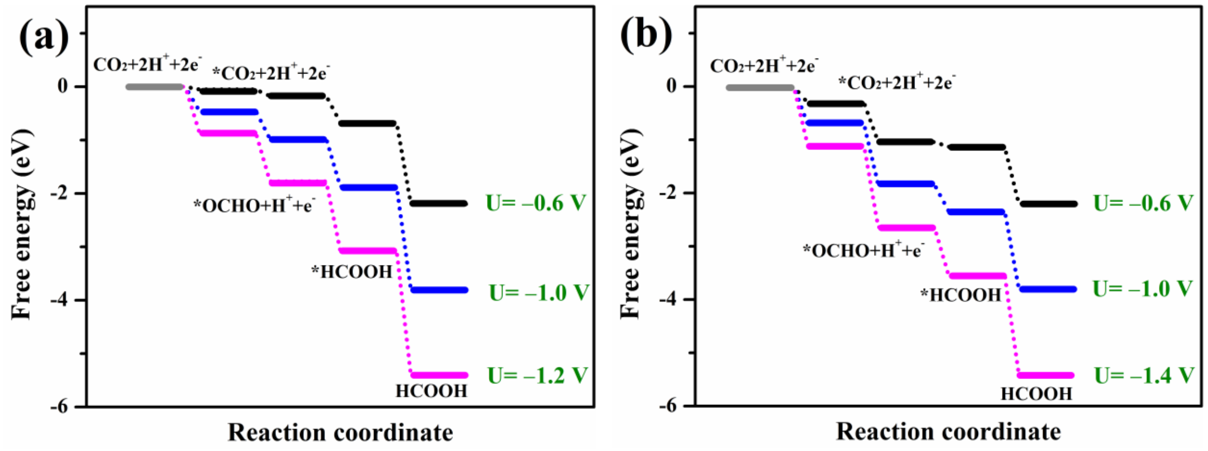

Fig. S20 Calculated free-energy diagrams at different external potentials (U= –0.6, –1.0 and –1.4 V) for CO_2 reduction to HCOOH on (**a**) Bi(012) and (**b**) Bi-B(012) surfaces

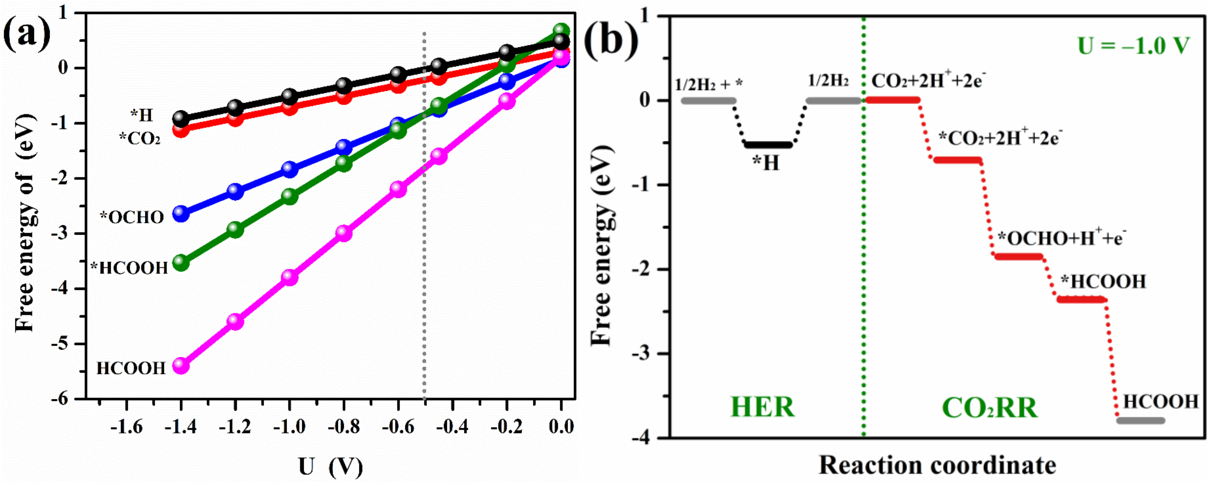

Fig. S21 Calculated free-energy diagrams of key intermediates for *H, *CO_2, *OCHO, *HCOOH and HCOOH (**a**) at different Us and (**b**) at U= –1.0 V as an example on Bi-B (012) surface

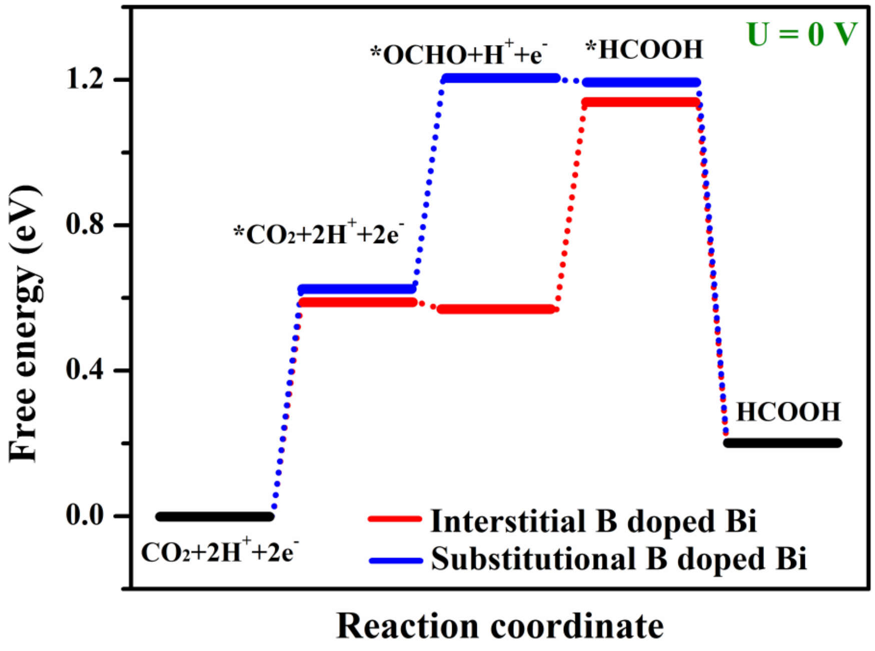

Fig. S22 Free energy diagrams of interstitial and substitutional B doped Bi with 1/18 ML configuration at U= 0 V

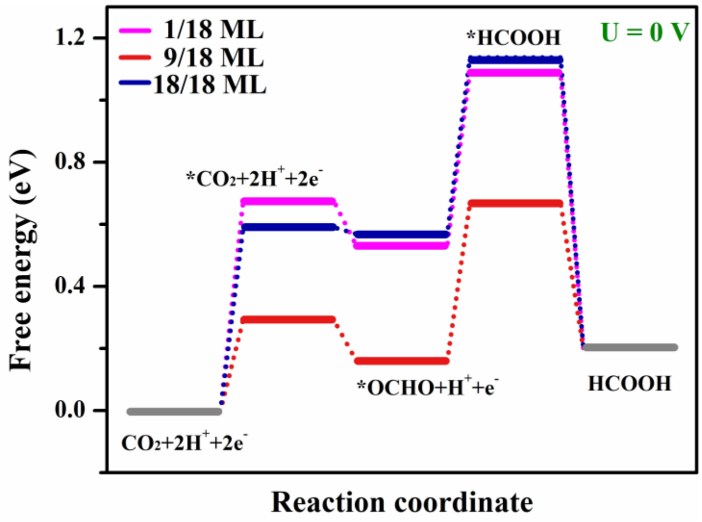

Fig. S23 Free energy diagrams of Bi with different B concentrations at U= 0 V

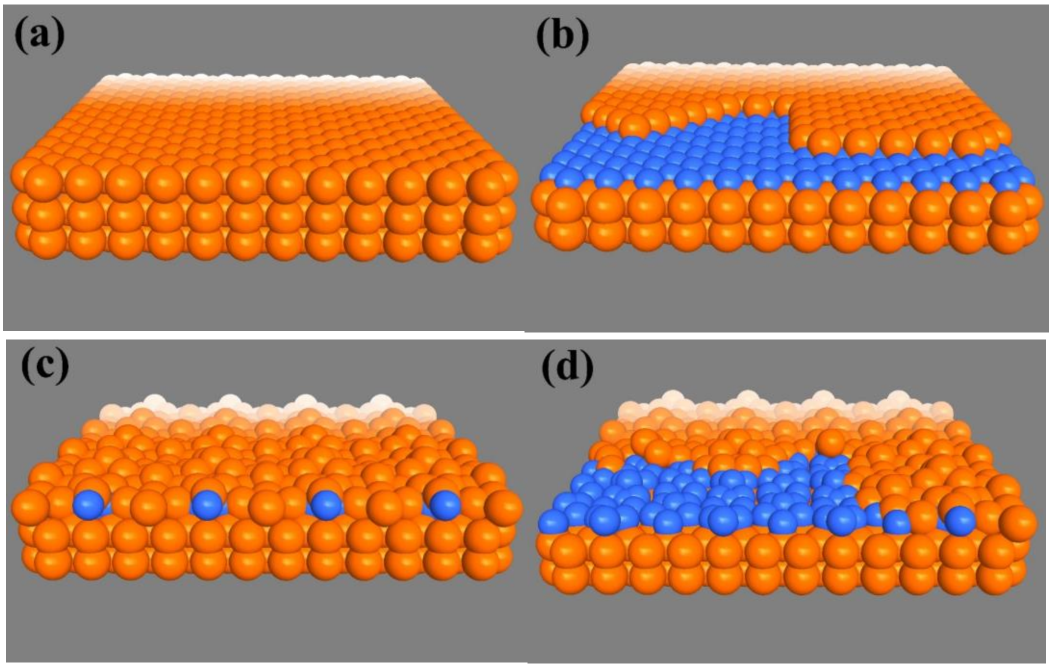

Fig. S24 (**a**, **b**) Initial and (**c**, **d**) after geometrical optimization DFT models of a B-doped Bi surface with a 18/18 ML concentration of boron

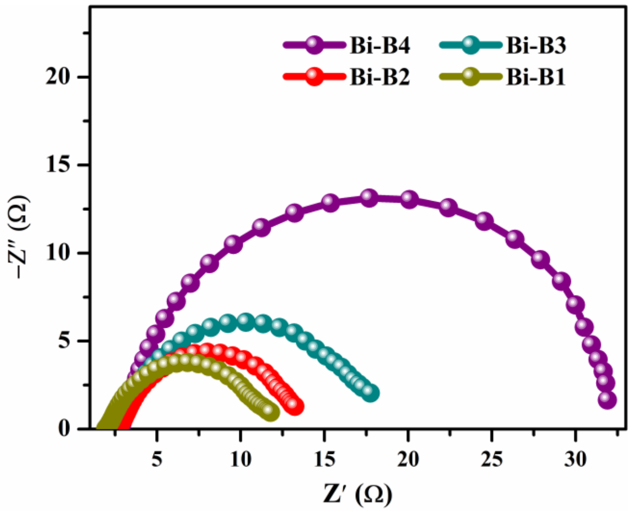

Fig. S25 Nyquist plots of different Bi-B samples

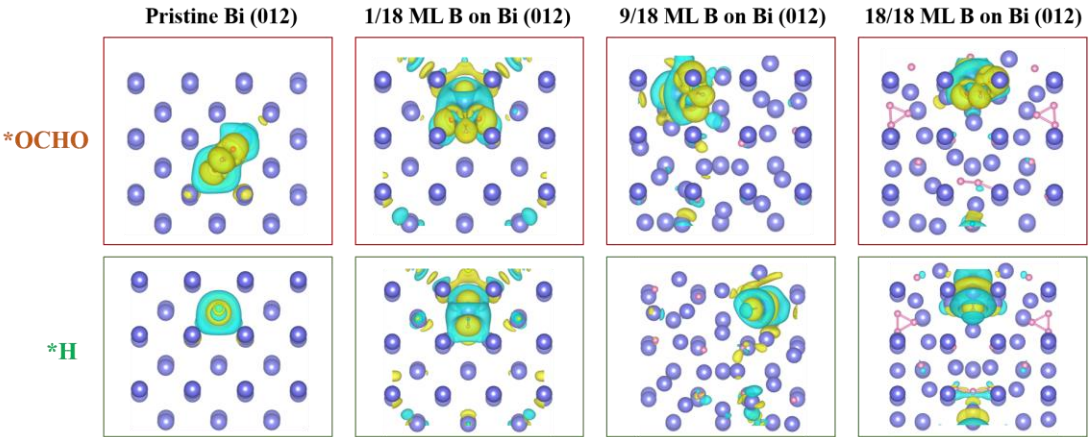

Fig. S26 Top view of differential charge densities of *OCHO and *H adsorbed on different surfaces, regions of yellow and cyan denote electron accumulation and depletion, respectively. Blue, pink, brown, red, and pale balls represent Bi, B, C, O, and H atoms, respectively. (The value of isosurface is 0.0005 e Å^{-3})

Table S1

Catalyst	Electrolyte ($KHCO_3$)	Potential range (FE>90%)	Potential range (FE>95%)	Refs.
Bismuthene	0.5 M	–0.73 V~ –1.18 V	–0.83 V~ –1.18V	[S1]
Bi nanosheet	0.1 M	–0.6 V~ –0.95 V	–0.77 V~ –0.87 V	[S2]
Bi Nanosheet	0.5 M	–0.7 V~ –0.8 V	–0.7 V	[S3]
Bi Nanosheet	0.1 M	-0.9 V	none	[S4]
nanoscale Bi	0.5 M	–0.97 V~ –1.17 V	–0.97 V	[S5]
Bi/rGO	0.1 M	–0.8 V ~ –0.9 V	–0.8 V	[S6]
dendritic Bi	0.5 M	–0.72 V~ –0.92 V	–0.82 V	[S7]
Bi nanotube	0.5 M	–0.78 V~ –1.2 V	–0.9 V~ –1.05 V	[S8]
Bi-NSS	0.1 M	–1.0 V~ –1.3 V	–1.1 V~ –1.2 V	[S9]
Bi@Bi_2O_3	0.5 M	–0.65 V~ –1.0 V	–0.72 V~ –0.9 V	[S10]
Bi NWs	–	–0.69 V ~ –0.99 V	–0.69 V	[S11]
NTD-Bi	0.5 M	−1.0 ~ –0.7V	–0.75 V ~ –0.9 V	[S12]
Bi-B2	0.5 M	–0.72 V~ –1.22 V	–0.75 V~ –1.13 V	This work

Supplementary References

[S1] C. Cao, D.D. Ma, J.F. Gu, X. Xie, G. Zeng et al., Metal-organic layers leading to atomically thin bismuthene for efficient carbon dioxide electroreduction to liquid fuel. Angew. Chem. Int. Edit. **59**(35), 15014-15020 (2020). https://doi.org/10.1002/anie.202005577

[S2] M.M. Zhao, Y.L. Gu, W.C. Gao, P.X. Cui, H. Tang et al., Atom vacancies induced electron-rich surface of ultrathin Bi nanosheet for efficient electrochemical CO_2 reduction. Appl. Catal. B Environ. **266**, 118625 (2020). https://doi.org/10.1016/j.apcatb.2020.118625

[S3] L. Yi, J. Chen, P. Shao, J. Huang, X. Peng et al., Molten salt assisted synthesis of bismuth nanosheets for long-term continuously electrocatalytic conversion CO_2 to formate. Angew. Chem. Int. Edit. **59**, 20112-20119 (2020). https://doi.org/10.1002/anie.202008316

[S4] F. Li, G.H. Gu, C. Choi, P. Kolla, S. Hong et al., Highly stable two-dimensional bismuth metal-organic frameworks for efficient electrochemical reduction of CO_2. Appl. Catal. B Environ. **277**, 119241 (2020). https://doi.org/10.1016/j.apcatb.2020.119241

[S5] P. Lamagni, M. Miola, J. Catalano, M.S. Hvid, M.A.H. Mamakhel et al., Restructuring metal–organic frameworks to nanoscale bismuth electrocatalysts for highly active and selective CO_2 reduction to formate. Adv. Funct. Mater. **30**(16), 1910408 (2020). https://doi.org/10.1002/adfm.201910408

[S6] Y.X. Duan, K.H. Liu, Q. Zhang, J.M. Yan, Q. Jiang, Efficient CO_2 reduction to HCOOH with high selectivity and energy efficiency over Bi/rGO catalyst. Small Methods **4**(5), 1900846 (2020). https://doi.org/10.1002/smtd.201900846

[S7] M.Y. Fan, S. Prabhudev, S. Garbarino, J.L. Qiao, G.A. Botton et al., Uncovering the nature of electroactive sites in nano architectured dendritic Bi for highly efficient CO_2 electroreduction to formate. Appl. Catal. B Environ. **274**, 119031 (2020). https://doi.org/10.1016/j.apcatb.2020.119031

[S8] K. Fan, Y.F. Jia, Y.F. Ji, P.Y. Kuang, B.C. Zhu et al., Curved surface boosts electrochemical CO_2 reduction to formate via bismuth nanotubes in a wide potential window. ACS Catal. **10**(1), 358-364 (2020). https://doi.org/10.1021/acscatal.9b04516

[S9] F.L. Meng, Q. Zhang, Y.X. Duan, K.H. Liu, X.B. Zhang, Structural optimization of metal oxyhalide for CO_2 reduction with high selectivity and current density. Chin. J. Chem. **38**(12), 1752-1756 (2020). https://doi.org/10.1002/cjoc.202000203

[S10] Y.R. Wang, R.X. Yang, Y.F. Chen, G.K. Gao, Y.J. Wang et al., Chloroplast-like porous bismuth-based core-shell structure for high energy efficiency CO_2 electroreduction. Sci. Bullet. **65**(19), 1635-1642 (2020). https://doi.org/10.1016/j.scib.2020.05.010

[S11] X.L. Zhang, X.H. Sun, S.X. Guo, A.M. Bond, J. Zhang, Formation of lattice-dislocated bismuth nanowires on copper foam for enhanced electrocatalytic CO_2 reduction at low overpotential. Energy Environ. Sci. **12**(4), 1334-1340 (2019). https://doi.org/10.1039/C9EE00018F

[S12] Q.F. Gong, P. Ding, M.Q. Xu, X.R. Zhu, M.Y. Wang et al., Structural defects on converted bismuth oxide nanotubes enable highly active electrocatalysis of carbon dioxide reduction. Nat. Commun. **10**, 2807 (2019). https://doi.org/10.1038/s41467-019-10819-4
